# The CD9/CD81 Tetraspanin Complex and Tetraspanin CD151 Regulate α3β1 Integrin-Dependent Tumor Cell Behaviors by Overlapping but Distinct Mechanisms

**DOI:** 10.1371/journal.pone.0061834

**Published:** 2013-04-17

**Authors:** Elisabeth Gustafson-Wagner, Christopher S. Stipp

**Affiliations:** 1 Department of Biology, University of Iowa, Iowa City, Iowa, United States of America; 2 Department of Molecular Physiology and Biophysics, University of Iowa, Iowa City, Iowa, United States of America; 3 Holden Comprehensive Cancer Center, University of Iowa, Iowa City, Iowa, United States of America; King's College London, United Kingdom

## Abstract

Integrin α3β1 potently promotes cell motility on its ligands, laminin-332 and laminin-511, and this may help to explain why α3β1 has repeatedly been linked to breast carcinoma progression and metastasis. The pro-migratory functions of α3β1 depend strongly on lateral interactions with cell surface tetraspanin proteins. Tetraspanin CD151 interacts directly with the α3 integrin subunit and links α3β1 integrin to other tetraspanins, including CD9 and CD81. Loss of CD151 disrupts α3β1 association with other tetraspanins and impairs α3β1-dependent motility. However, the extent to which tetraspanins other than CD151 are required for specific α3β1 functions is unclear. To begin to clarify which aspects of α3β1 function require which tetraspanins, we created breast carcinoma cells depleted of both CD9 and CD81 by RNA interference. Silencing both of these closely related tetraspanins was required to uncover their contributions to α3β1 function. We then directly compared our CD9/CD81-silenced cells to CD151-silenced cells. Both CD9/CD81-silenced cells and CD151-silenced cells showed delayed α3β1-dependent cell spreading on laminin-332. Surprisingly, however, once fully spread, CD9/CD81-silenced cells, but not CD151-silenced cells, displayed impaired α3β1-dependent directed motility and altered front-rear cell morphology. Also unexpectedly, the CD9/CD81 complex, but not CD151, was required to promote α3β1 association with PKCα in breast carcinoma cells, and a PKC inhibitor mimicked aspects of the CD9/CD81-silenced cell motility defect. Our data reveal overlapping, but surprisingly distinct contributions of specific tetraspanins to α3β1 integrin function. Importantly, some of CD9/CD81's α3β1 regulatory functions may not require CD9/CD81 to be physically linked to α3β1 by CD151.

## Introduction

Integrins, the major family of cellular receptors for extracellular matrix proteins, comprise 18 α and 8 β subunits, which assemble into 24 known αβ heterodimers with different ligand binding specificities [1]. Gene targeting studies in mice have revealed that integrins have essential functions in a wide array of developmental and homeostatic processes, ranging from embryo implantation and placenta formation early in development, to blood clotting and immunocyte function in adult animals [2]. Within the integrin family, the laminin-binding integrins, α3β1, α6β1, α6β4, and α7β1, constitute a distinct subfamily. These integrins play essential roles in the morphogenesis and maintenance of skin, kidney, and lung epithelia (α3 and α6 integrins) and muscle (α7 integrin) by binding to laminin isoforms in the basement membranes underlying these tissues [2]–[4]. In addition to ligand preference, the laminin-binding integrins share other biochemical similarities, including palmitoylation of the α3, α6, and β4 integrin subunit cytoplasmic tails [5], and physical interactions with tetraspanin proteins in the cell membrane [6]–[8].

Tetraspanins are a family of 33 proteins in mammals that are characterized by 4 transmembrane domains, cytoplasmic amino and carboxyl termini, and one small and one large extracellular loop, which contains a characteristic cysteine motif. Tetraspanins interact with themselves (both homotypically and heterotypically) and with a subset of other integral membrane proteins, including integrins, to assemble multi-protein complexes within dynamic membrane domains termed tetraspanin-enriched membrane microdomains (TEMs). Localization of the laminin-binding integrins to TEMs may provide access to a distinct array of cytoplasmic signaling proteins, including PI 4-kinase, ERM proteins, and classical PKC isoforms [6], [9]–[12].

The laminin-binding integrins have been extensively studied in the context of tumor cell biology because of their potent, context-dependent functions in regulating tumor formation, progression, invasion, and metastasis [6]. Where studied, α7 integrin functions as a suppressor of tumor growth and metastasis in a variety of tumor types [13], [14]. In contrast, α6β4 integrin promotes metastatic progression in breast and skin carcinoma [15]–[18], and α6β1 integrin exerts pro-survival and pro-metastatic functions in prostate carcinoma [19]–[21]. For α3β1 integrin the picture is complex. While α3β1 can promote breast cancer tumorigenesis in vivo [22], it is sometimes lost during prostate cancer progression [23], and forced α3β1 expression can suppress the growth of rhabdosarcoma in vivo [24] and block skin carcinoma progression [25]. Thus, in order to predict whether α3β1 will exert a pro- or anti-metastatic influence in specific cases, more insight into the molecular mechanisms of α3β1 function in tumor cells is required.

Pro-metastatic functions for α3β1 integrin in breast cancer may involve multiple mechanisms, including promoting (i) Cox-2 expression, (ii) matrix metalloproteinase MMP-9 secretion, (iii) tumor cell crosstalk with endothelial cells, and (iv) Src, FAK, and Rac activation [22], [26]–[29]. The activation of a Src/FAK-Rac signaling pathway may underlie α3β1's ability to promote unusually rapid migration on its laminin ligands, laminin-332 (LM-332) and laminin-511. Integrin α3β1-dependent motility on these ligands can be as much as ∼4–5 fold more rapid than motility on non-α3β1 ligands, such as fibronectin or collagen I [30]–[33].

Many α3β1 functions in breast cancer cells may depend strongly on its association with tetraspanin proteins. Silencing of tetraspanin CD151, a major α3β1 integrin partner, has several effects on tumor cell behavior in the MDA-MB-231 breast carcinoma model, including (i) reduced migration toward LM-332 in transwell assays [34], (ii) reduced Matrigel invasion towards EGF, reduced EGF-induced spreading on laminin-111 (LM-111), and reduced subcutaneous or orthotopic growth upon implantation in nude mice [35], (iii) reduced subcutaneous tumor growth and reduced scattering in 3D Matrigel in response to endothelial cell conditioned medium [28], (iv) reduced TGFβ1-induced cell scattering in 3D Matrigel, and reduced lung colonization upon tail vein injection in nude mice [36], and (v) reduced adhesion on LM-332 and LM-111, impaired cable formation on 3D Matrigel, and impaired spreading on LM-111 in response to phorbol ester [37].

While the findings described above support the view that CD151 makes critical contributions to the functions of laminin-binding integrins in breast cancer cells, most of these studies either focused primarily on α6 integrin, or utilized substrates such as Matrigel for which both α3 and α6 integrins can make functional contributions. Thus, it remains unclear whether the mechanisms by which tetraspanins regulate α3β1 integrin in breast carcinoma cells are identical to the mechanisms by which they regulate α6 integrins. Moreover, CD151 loss-of-function phenotypes are often interpreted in terms of CD151's ability to link its integrin partners to other TEM-resident proteins, but the loss-of-function phenotypes of other TEM resident proteins have generally not been carefully compared to those of CD151 in side-by-side studies.

To begin to clarify which aspects of CD151's α3β1 integrin-regulatory functions may depend on which other TEM-resident proteins, we created breast carcinoma cells with profound RNAi-mediated silencing of CD9 and CD81, two closely related tetraspanins that engage in similar biochemical interactions [38]–[42] and which strongly depend on CD151 for association with α3β1 [30], [35], [43], [44]. We then compared the phenotypes of our CD9/CD81-silenced cells directly to the phenotypes of CD151-silenced cells. Our data reveal that the CD9/CD81 complex and CD151 have overlapping but distinct functions in regulating α3β1-dependent behaviors in the MDA-MB-231 breast cancer model. Surprisingly, several CD9/CD81 functions, including promoting α3β1's association with PKCα and promoting α3β1-dependent tumor cell migration on LM-332, may not require CD151-dependent complex formation with α3β1 in this system.

## Materials and Methods

### Antibodies, Extracellular Matrix Proteins, and Pharmacological Reagents

Antibodies used in this were anti-CD9, ALB6, (Meridian Life Science); anti-CD81, M38 [45], anti-CD151, 5C11 [46]; anti-α3 integrin, A3-X8 and A3-IIF5 [47]; anti-α6 integrin, GoH3 (eBioscience); anti-CD55, BRIC-216 (Millipore); rabbit monoclonal anti-PKCα (Abcam ab32376); rabbit polyclonal anti-PKCα (Santa Cruz sc208); anti-β-actin, AC-15 (Sigma); and polyclonal anti-α3 integrin cytoplasmic tail antibody, A3-CYT [43]. Secondary reagents used were Alexa 488-goat anti-mouse, and Alexa 680-goat anti-rabbit (both from Invitrogen), Cy2 goat anti-mouse (Jackson ImmunoResearch), and IRDye 800-goat anti-mouse (Rockland Immunochemicals, Inc).

Rat tail collagen I and Matrigel were from BD Bioscience. LM-332 was purified from SCC25 squamous carcinoma cell-conditioned medium as previously described [30]. Phorbol-12-myristate-13-acetate (PMA) was from Sigma, and classical PKC isoform inhibitor, Gö6976, was from Tocris Bioscience.

### Cell culture, RNAi and Retroviral Transduction

MDA-MB-231 breast carcinoma (ATCC), A431 epithelial carcinoma (available from ATCC; obtained from the lab of Martin Hemler, Dana-Farber Cancer Institute) and GP2-293 retroviral packaging cells (Clontech) were cultured in high glucose DME medium supplemented with 10% fetal bovine serum, 2 mM L-glutamine, 100 U/ml Penicillin, and 100 ug/ml Streptomycin (Invitrogen). For RNAi, double-stranded oligonucleotides encoding short hairpin RNAs (shRNAs) targeting the human CD9, or CD81, or CD151 mRNAs were annealed and then cloned into the pSIREN-RetroQ retroviral vector (BD Biosciences) as described [30]. The shRNA targeting sequences used were CD9: 5′-AGAGCAUCUUCGAGCAAGAAA-3′; CD81: 5′-GGUCAUCCUG-UUUGCCUGUGA-3′, and CD151 sh3: 5′-AGUACCUGCUGUUUACCUACA-3′. To facilitate simultaneous knockdown of CD9 and CD81, the puromycin selectable marker of pSIREN-RetroQ was replaced with a hygromycin resistance gene using recombinant PCR, and the CD9 targeting construct was cloned into this modified pSIREN-RetroQ-hygro vector. Retroviral particles produced in GP2-293 cells were used to transduce MD-MBA-231 or A431 cells and stable transductants were selected with puromycin, hygromycin, or both. Stably transduced cells were sorted by flow cytometry for loss of CD9, CD81, or CD151, and maintained as polyclonal populations.

For CD9 or CD81 re-expression in the CD9/CD81 doubly-silenced cells, recombinant PCR was used to generate CD9 and CD81 cDNAs containing two silent mutations within the RNAi target sequences. These constructs, named CD9RX and CD81RX, and were cloned into the pLXIZ retroviral expression vector for transduction into CD9/CD81-silenced cells, followed by zeomycin selection. Re-expression of CD9 or CD81 in the CD9/CD81-silenced cells was confirmed by flow cytometry. CD151 re-expression in the CD151-silenced cells was accomplished in the same manner, using our previously published CD151RX construct [30].

### Cell Spreading Assay

To assess short term cell spreading, 5×10^5^ cells were plated in a T25 flask on day 1. On day 2, cells were starved overnight in serum-free medium (SFM), which was DMEM with 5 mg/ml cell culture grade BSA (Sigma cat. #A1470) and 25 mM HEPES pH 7.2. On day 3, cells were treated with trypsin-EDTA for 3 minutes and harvested in SFM supplemented with 0.1 mg/ml soybean trypsin inhibitor and 20 µg/ml DNAse I (Worthington Biochemical, Lakewood, NJ). Cells were collected by centrifugation, resuspended in SFM, and plated at 2.5×10^4^ cells/cm^2^ in 35 mm dishes or glass coverslips that had been coated with 1 µg/ml LM-332 or 20 µg/ml collagen I. After 30 min, cells were fixed, and photographed on a Leica DMIRE2 inverted microscope using a 20× phase objective. NIH Image J software [48] was used to measure the spread area of at least 25 cells per cell type. Specific spreading was calculated by subtracting the mean area of unspread cells (fixed immediately after plating) from the spread cell areas measured at the end of the assay, as in [49]. In some experiments, α3 integrin function-blocking antibody, A3-IIF5, or α6 integrin function blocking antibody, GoH3, was added to cells at 10 µg/ml for 10 min prior to plating.

### Time-lapse Cell Motility Assay

Cells were serum-starved and harvested as described for the cell spreading assays above and then plated in 35 mm glass bottom dishes (MatTek Corp.) that had been coated with 1 µg/ml LM-332 or 20 µg/ml collagen I. Cells were allowed to attach and spread and then imaged using a stage incubator and video-microscope system described previously [30]. Images were collected every 2 minutes for 3 hours, unless otherwise specified. Image J software was used to record XY coordinates of at least 20 cell centroids per movie in 4 minute intervals. Only cells that remained in view for at least 1 h were included in the analysis. Custom Java software was used to calculate mean velocity, net distance traveled, and persistence (net distance traveled/total distance traveled). In some assays, function blocking antibodies or the PKC inhibitor Gö6976 were added after 2 h, and cell motility was monitored for an additional 2–3 h. Quantification of rear cell membrane projections (tails) was performed by measuring (i) the total number of projections that lasted at least 4 min in at least 25 cells per cell type, and (ii) the total duration for each of the observed tails. One way ANOVA with Tukey post test was used to assess statistical significance of differences among cell types.

### Immunoprecipitation and Immunoblotting

Cells were lysed in 20 mM HEPES pH 7.2, 150 mM NaCl, 1 mM MgCl_2_ with 1% detergent, protease inhibitors (2 mM PMSF, 10 µg/ml aprotinin, 5 µg/ml leupeptin, and 5 µg/ml E-64) (Roche Diagnostics) and HALT phosphatase inhibitors (ThermoFisher Pierce). Detergents used were (i) Brij 99 (Acros Organics), (ii) a 1∶1 mixture of Brij 96V (Fluka) and Brij 99, or (iii) Brij 58 (Acros Organics). Lysate protein concentrations were normalized on the basis of a Red 660 protein assay (G Biosciences). Primary antibodies were added at 5 µg/ml, and immune complexes were collected with protein G sepharose (ThermoFisher Pierce) and analyzed by SDS-PAGE and immunoblotting. Blots were blocked with AquaBlock (East Coast Bio), and developed with primary antibody, followed by IRDye 800-goat anti-mouse or Alexa 680-goat anti-rabbit secondary antibodies, and scanning with an Odyssey infrared imaging system (LI-COR Biosciences). For PKCα association studies, cells were stimulated with 100 nM PMA for 30 min prior to lysis and co-immunoprecipitation experiments, as previously described [50]. Immunoprecipitates were analyzed by SDS-PAGE under reducing conditions. To estimate the fraction of total cellular PKCα associated with α3β1 integrin, PKCα band intensities were quantified with Image Studio Lite software (LI-COR Biosciences), and the amount of PKCα co-precipitating with α3 integrin was divided by the amount of total PKCα detected in lysates, correcting for the volume of lysate input into the immunoprecipitation and the volume of lysate loaded on the gel.

### Adhesion Assay

Cells were serum-starved, harvested, and resuspended in SFM, as described above for the cell spreading assay. Next, 1×10^4^ cells per well were plated in 96 well plates coated with 1 µg/ml LM-332, 20 µg/ml collagen I, or 10 mg/ml heat-inactivated BSA (negative control). After 45 min non-adherent cells were removed by rinsing, and adherent cells were fixed, stained with crystal violet, and quantified, as previously described [30].

### Cell Growth in 3D Matrigel

Cells were resuspended in growth factor reduced Matrigel and plated at 5000 cells/well in a 24 well plate over a pre-polymerized cushion of Matrigel. After polymerizing for 30 min at 37°C, the wells were overlaid with standard growth medium. Colony growth was quantified by measuring the area of at least 25 colonies for each cell type at 1, 4, and 5 weeks post-plating, using Image J software. ANOVA with post-hoc t tests was used to determine the statistical significance of observed differences.

## Results

### Efficient silencing of tetraspanins CD9 and CD81 in MDA-MB-231 breast cancer cells

Tetraspanins CD9 and CD81 strongly associate with one another and with a similar spectrum of tetraspanin partners, including the immunoglobin superfamily proteins EWI-2 and EWI-F/CD9P-1 [38]–[42]. After preliminary experiments, in which silencing CD9 or CD81 alone in MDA-MB-231 breast cancer cells produced only modest phenotypic changes (Fig. S1), we established cells with stable silencing of both tetraspanins (CD9/81si cells). Flow cytometry revealed that cell surface CD9 expression was reduced ∼90% and cell surface CD81 expression by ∼80% in the CD9/81si cells, as compared to parental MDA-MB-231 cells (Table 1). For specificity controls, we selectively restored CD9 (CD9RX cells) or CD81 (CD81RX cells) using expression constructs designed to evade CD9 or CD81 RNAi (Table 1). All cell lines were maintained as stable, polyclonal populations.

**Table 1:** Flow cytometry of MDA-MB-231 & A431 cell lines.

Cell Type	CD9 expression (% WT)	CD81 expression (%WT)	CD151 expression (%WT)	α3 integrin expression (%WT)
**MDA-MB-231 lines**				
**WT**	100	100	100	100
**CD9si**	**24**	134	111	76
**CD81si**	145	**4**	132	97
**CD9/CD81si**	**11±3** a	**20±6** a	99±12^b^	69±6^b^
**CD9RX**	**107**	25	103	64
**CD81RX**	18	**116**	190	73
**CD151si**	104±18^b^	123±8^b^	**0.8±0.4** b	107±6^b^
**CD151RX**	88±12^b^	69±4^b^	**121±13** b	117±27^b^
**A431 lines**				
**WT**	100	100	ND^c^	ND
**CD9/CD81si**	**5**	**11**	ND	ND

a (n = 11 separate determinations).

b (n = 3 separate determinations).

c ND, not determined.

### Delayed spreading of CD9/CD81-deficient cells on LM-332

To begin to explore how the CD9/CD81 complex regulates α3β1 integrin function, we first determined the contributions of the two major MDA-MB-231 laminin receptors, α3 and α6 integrins, to cell attachment and spreading on LM-332. Functional blockade of α3 integrin, but not α6 integrin, virtually abolished cell attachment and spreading on LM-332 (Fig. S2A). For cells that had already attached and begun migrating on LM-332, addition of an anti-α3 integrin function blocking antibody caused rapid cell rounding and a cessation of motility, while an anti-α6 integrin antibody had no effect within 2 h after addition (Fig. S2B). These data established that MDA-MB-231 cell responses to LM-332 are strongly α3β1 integrin-dependent.

**Figure 1:**
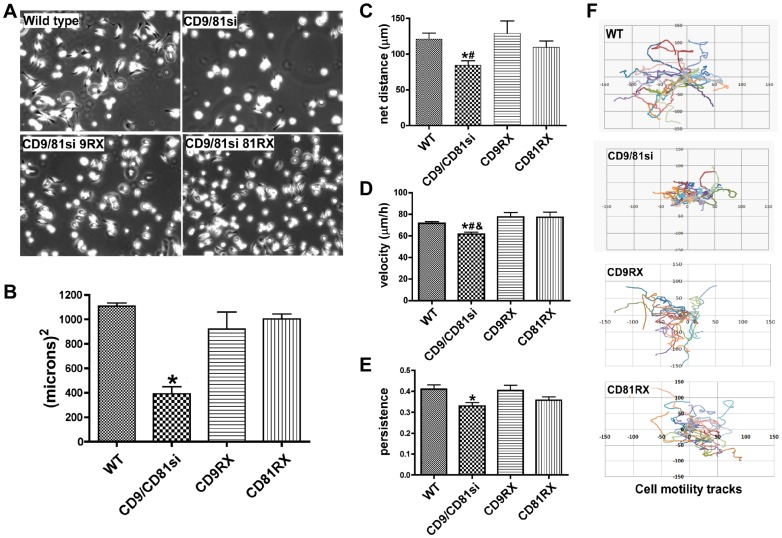
Cell spreading and directed migration is impaired in CD9/CD81-depleted MDA-MB-231. (**A**) MDA-MB-231 Wild type, CD9/CD81si, CD9RX, and CD81RX cells were plated on LM-332-coated glass coverslips, and cell spreading was imaged 30 min later by phase contrast microscopy. (**B**) The area of cell spreading for each cell type was calculated by subtracting the mean area of cells imaged immediately after plating from the mean area of cells after 30 min of spreading. Values are means ± s.e.m.; n = 3 trials with at least 25 cells of each cell type per trial; *P<0.01 (CD9/CD81si vs. WT, CD9RX and CD81RX, ANOVA with Tukey post-test). (**C–E**) MDA-MB-231 wild type, CD9/CD81si, CD9RX or CD81RX cells were plated on LM-332, allowed to attach and fully spread, and then monitored by time-lapse microscopy for 3 h. Net distance traveled (**C**), migration velocity (**D**), and persistence (**E**) were measured using ImageJ. Values are means ± s.e.m.; n = 9 trials (WT and CD9/CD81si cells) or 4 trials (CD9RX and CD81RX cells), with 25–50 cells of each type per trial. CD9/CD81si showed significantly reduced migration parameters compared to wild type (*P<0.05), CD9RX (^#^P<0.10), and CD81RX (^&^P<0.01) cells, ANOVA with Tukey post-test. (**F**) The graphs show motility tracks representing cell movement from point of origin over 3 h for 25 cells of each type.

Spreading of CD9/CD81si cells on LM-332 was significantly delayed compared to the parental cells, as visualized 30 min after plating (Fig. 1A). Restoring either CD9 or CD81 expression in the CD9/81si cells restored spreading to near normal levels (Fig. 1A). Quantification of cell spreading area (the total spread area minus the mean area of unspread cells) confirmed an ∼60% reduction for the CD9/81si cells compared to parental cells, which was reversed in the CD9RX and CD81RX cells (Fig. 1B). The ability of either CD9 or CD81 re-expression to reverse the spreading defect of the CD9/CD81si cells was expected since silencing either tetraspanin individually was not sufficient to uncover a spreading defect (Fig. S1). Thus, the presence of either CD9 or CD81 is required for efficient α3β1 integrin-dependent spreading at early time points. At 60 min after plating, all cell types were equally well spread (not shown), thus the loss of CD9 and CD81 delayed spreading, but did not ultimately prevent it.

### Impaired directed migration for CD9/CD81-deficient cells on LM-332

We used time-lapse video-microscopy to study post-attachment cell behavior on LM-332. Compared to the parental MDA-MB-231 cells, CD9/CD81si cells showed a significant reduction in the net distance traveled during the observation period. (Fig. 1C). Contributing to this reduction in net migration distance was both a modest reduction in the absolute migration velocity of CD9/CD81si cells (Fig. 1D), and a somewhat larger reduction in the directional persistence of migration (Fig. 1E). Persistence, a measure of the extent to which cells continue to migrate in the same direction over time, is defined here as the ratio of net distance traveled to total distance traveled. Re-expression of CD9 or CD81 in the CD9/CD81si cells partially or completely restored cell migration parameters to wild type levels (Fig. 1C–E). The altered migration pattern of the CD9/CD81si cells is readily appreciated when their cell tracks are plotted (Fig. 1F). Compared to parental and rescue cells, the CD9/CD81si cells migrated shorter distances before turning and migrating in another direction, resulting in more highly convoluted tracks. Together, the cell motility data in Figure 1 revealed that α3β1 integrin-dependent directed cell migration is defective in cells depleted of CD9 and CD81.

### Front-rear morphology is altered in CD9/CD81-silenced cells

**Figure 2:**
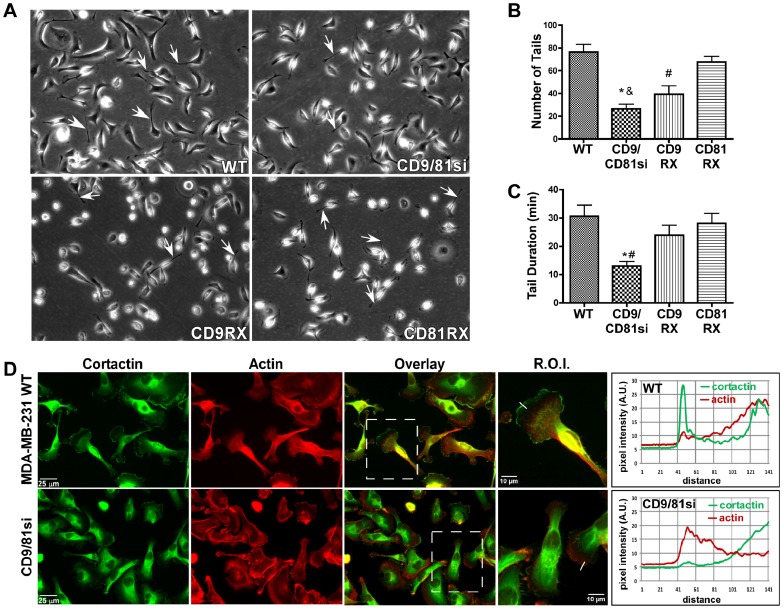
Altered front-rear cell morphology in CD9/CD81-silenced cells. (**A**) Wild type MDA-MB-231 cells migrating on LM-332 frequently developed long protrusions at the rear of the cell (white arrows), which were less frequently observed in the CD9/CD81si cells. (**B**) The number of protrusions per cell lasting 4 min or longer during a 3 h video was quantified for wild type, CD9/CD81si, CD9RX, and CD81 RX cells. Values are means ± s.e.m.; n = 3 trials with 25 cells/trial. CD9/CD81si cells developed significantly fewer tails than wild type (*P<0.01) or CD81RX cells (^&^P<0.01); CD9RX cells also had somewhat fewer tails than wild type (^#^P<0.01), ANOVA with Tukey post-test. (**C**) The total duration of rear protrusion tails that developed in each cell type. Values are means ± s.e.m.; n = 3 trials with 25 cells/trial. CD9/CD81si cell tail duration was significantly less than wild type (*P<0.05) and CD81RX (^#^P<0.05). (**D**) Cortactin and Phalloidin staining of MDA-MB-231 wild type and CD9/CD81si cells. Cells were plated on LM-332-coated coverslips for 1 h, then fixed, permeabilized, and stained with anti-cortactin antibody (green) and with phalloidin, to reveal F-actin (red). Boxed areas in the overlay panels are shown as zoomed regions of interest (R.O.I.). Lines drawn across the leading edges of 2 representative cells in the overlay panels were used for pixel intensity scans of cortactin staining (green trace) and F-actin staining (red trace), graphed in arbitrary units. Intensity scans ran from outside the cells into the cell interiors, and are graphed left to right.

The defective directed migration of the CD9/CD81si cells may be related to altered front-rear morphology in these cells. Parental MDA-MB-231 cells migrating on LM-332 frequently developed long retraction tails at the rear of the cell (Fig. 2A–C). Both the frequency and duration of retraction tails was dramatically reduced in the CD9/CD81si cells (Fig. 2A–C). Re-expressing CD81, and to a lesser extent, CD9, restored retraction tail frequency and duration to more normal values (Fig. 2A–C). In addition to altered retraction tail morphology, CD9/CD81si cells also displayed altered localization of cortactin. Cortactin is normally recruited to the leading edge of lamellipodia, where it promotes the formation of new adhesions and lamellipodial persistence [51]. In parental cells, cortactin typically co-localized with F-actin in a thin zone at the very leading edge of lamellipodia (Fig. 2D, top panels). In contrast, in the CD9/CD81si cells, although thick zones of F-actin were often present at the lamellipodial leading edge, cortactin staining was often absent from this region (bottom panels), suggesting that leading edge identity, actin dynamics, or adhesion formation may be altered in CD9/CD81si cells. The altered relationship between cortactin and F-actin localization in the CD9/CD81si cells can be appreciated from pixel intensity scans intersecting the lamellipodia of each cell type (Fig. 2D, graphs). Quantification revealed that ∼84% of parental cells stained positive for leading edge cortactin, while only ∼24% of CD9/CD81si cells were positive for leading edge cortactin (p<0.0001, Fisher's exact test; Fig. S3). Thus, CD9/CD81si cells displayed morphological and/or molecular differences at both the leading and trailing edges of migrating cells.

### The CD9/CD81 complex and tetraspanin CD151 regulate overlapping but distinct aspects of α3β1 function in MDA-MB-231 cells

**Figure 3:**
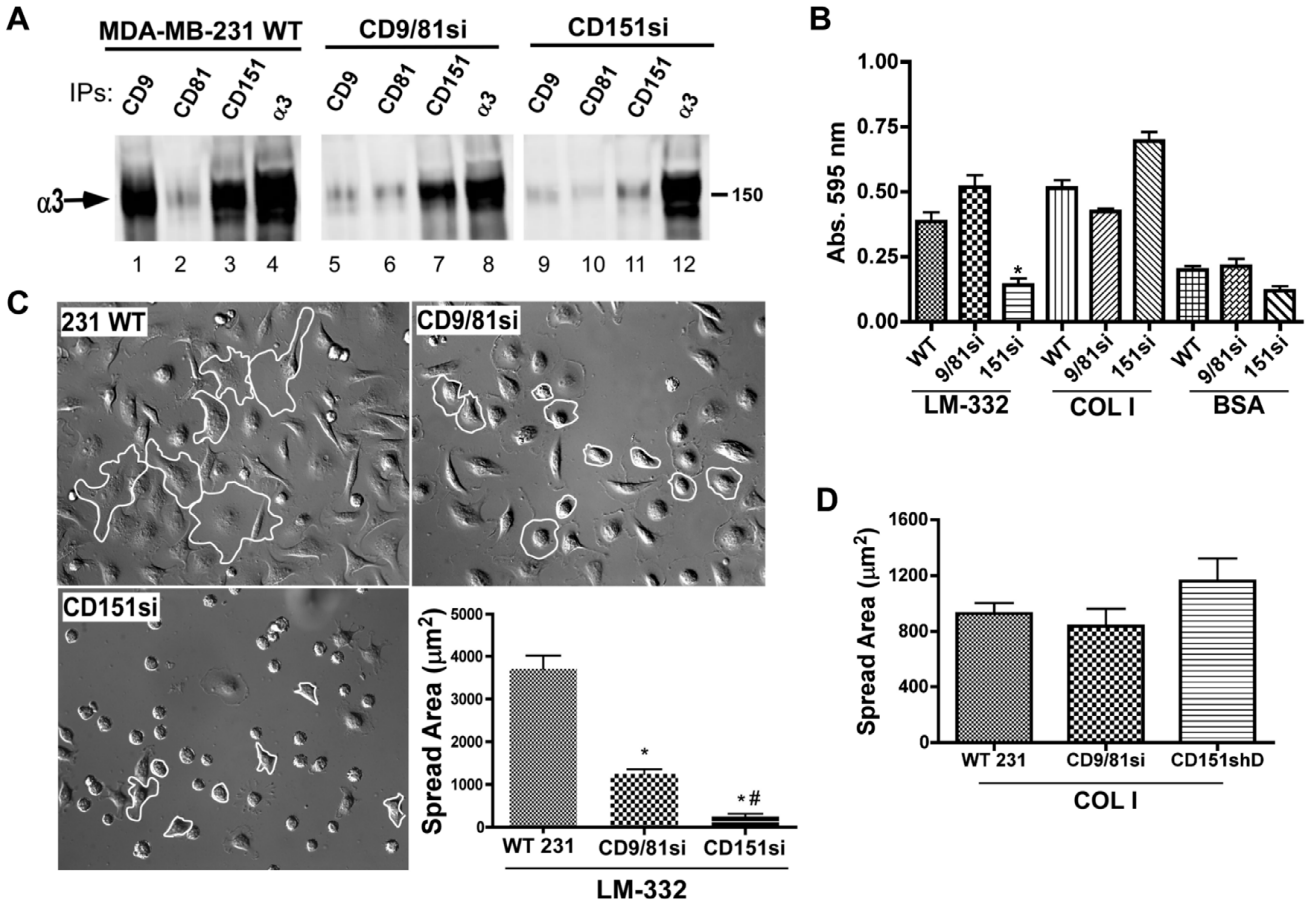
CD151 promotes α3 integrin-CD9 association, and is required for normal adhesion and initial cell spreading on LM-332. (**A**) MDA-MB-231 wild type, CD9/CD81si, and CD151si cells were lysed in 1% Brij 96V/Brij 99 (a 1∶1 mixture of both detergents), and CD9, CD81, CD151, or α3 integrin were immunoprecipitated (IPs), followed by blotting for the α3 integrin subunit. Note that α3 integrin-CD9 association is almost completely abolished in the CD151-silenced cells, but that α3-CD151 association is maintained in the CD9/CD81-silenced cells. (**B**) Wild type, CD9/CD81si, and CD151si MDA-MB-231 cells were plated in wells coated with LM-332, collagen I (COLI), or BSA for 30 min. Non-adherent cells were removed, and remaining cells were fixed and quantified by staining with crystal violet. Values are means ± s.e.m.; n = 4 wells/cell type. CD151si cells adhered less well than wild type or CD9/CD81si cells to LM-332 (*P<0.001), ANOVA with Tukey post-test. (**C**) Wild type, CD9/CD81si, and CD151si cells were plated on LM-332-coated coverslips and fixed after 45 min. Cell spreading area was measured as in Fig. 1B. Representative cells of each type are outlined in white to emphasize differences in spread area. Values graphed are means ± s.e.m.; n = 3 trials, with at least 25 cells of each type per trial. CD9/CD81si and CD151si cell spread area was less than that of wild type cells (*P<0.001), and CD151si cell spread area was less than CD9/CD81si cell spread area (^#^P<0.001), ANOVA with Tukey post-test. (**D**) Wild type, CD9/CD81si, and CD151si cells showed no significant differences in spreading on collagen I-coated coverslips.

Several previous studies have established that tetraspanin CD151 engages in stable, direct interactions with alpha subunits of laminin-binding integrins [46], [52]–[54] and promotes laminin-binding integrin association with other components of TEMs, including CD9 and CD81 [30], [35], [44], [55]. To compare the function of CD151 to that of the CD9/CD81 complex in regulating α3β1 integrin, we established CD151-silenced MDA-MB-231 cells (CD151si cells). Flow cytometry revealed near total silencing of CD151 in these cells, with minimal impact on the expression levels of CD9, CD81, or α3 integrin (Table 1). Co-immunoprecipitation experiments showed that a substantial fraction of the total α3 integrin (as measured by direct α3 immunoprecipitation) was recovered in either CD9 or CD151 immunoprecipitations (Fig. 3A, compare lanes 1&3 to lane 4). Much less α3 was detected co-precipitating with CD81 (Fig. 3A, lane 2), but we suspect that this may be due to epitope shielding of CD81 in mild detergent lysates (EGW & CSS unpublished observation). Silencing CD9 and CD81 virtually abolished recovery of α3 integrin in a CD9 immunoprecipitate, as expected (Fig. 3A, lane 5), but had little effect on the association of α3 with CD151 (Fig. 3A, lane 7). In contrast, in the CD151si cells, the CD9-α3 association was disrupted (Fig. 3A, lane 9) in addition to the anticipated loss of α3 recovery in a CD151 immunoprecipitate (Fig. 3A, lane 12). Collectively, these data show that CD151 is required to promote α3-CD9 association, but CD9 is not required to promote α3-CD151 association. This result is in accord with our prior study showing that CD151 may act as a direct physical linker of laminin-binding integrins to other TEM-resident proteins [43].

To compare the functional impact of CD151 depletion to that of CD9/CD81 depletion, we performed additional adhesion, spreading, and motility experiments on LM-332. CD151si cells displayed a dramatic loss of adhesion in short term assays on LM-332, while CD9/CD81si cells adhered equally as well as the parental MDA-MB-231 cells (Fig. 3B). All cell types adhered well to the α2β1 integrin ligand, collagen I, with CD151si cells perhaps even showing enhanced adhesion compared to parental cells (Fig. 3B). In cell spreading assays on LM-332, CD9/CD81si cells again displayed delayed spreading (Fig. 3C), as previously observed in Fig. 1A. However the CD151si cells showed an even more profound spreading defect on LM-332 than the CD9/CD81si cells (Fig. 3C). By ∼1 h post-plating, all 3 cell types had spread equally well (not shown), indicating that the spreading defect on LM-332 is transient, as previously observed. In contrast to the results on LM-332, all three cell types displayed similar rapid spreading on collagen I (Fig. 3D).

**Figure 4:**
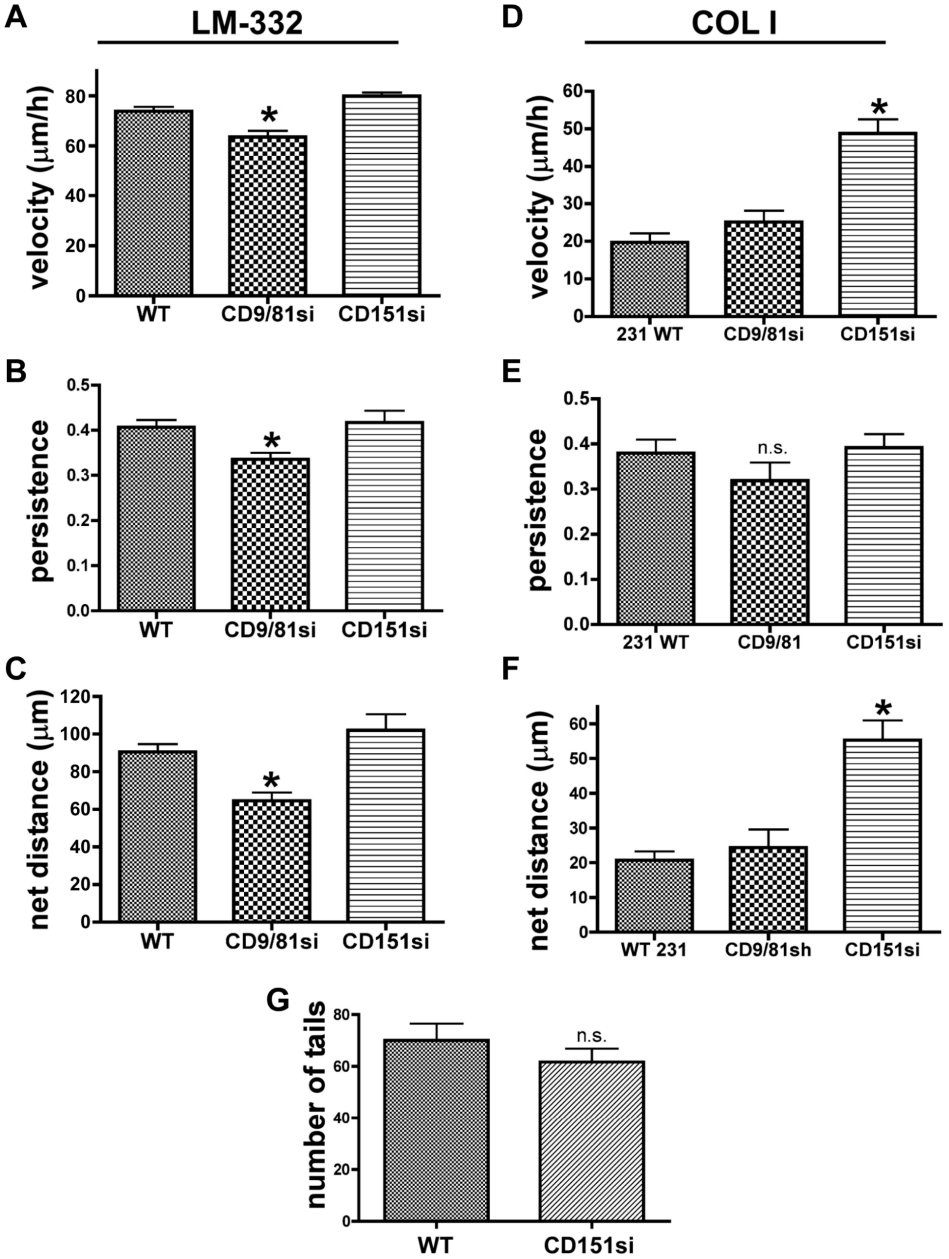
CD151 depletion does not impair MDA-MB-231 cell migration on LM-332. MDA-MB-231 wild type, CD9/CD81si, or CD151si cells were plated on LM-332 or collagen I-coated glass bottom dishes. After cells had attached and spread, motility was monitored by time-lapse microscopy, as in Fig. 1. (**A**) CD9/CD81si cells, but not CD151si cells, displayed reduced migration velocity on LM-332 compared to wild type cells (*P<0.01). (**B**) CD9/CD81si cells, but not CD151si cells, displayed reduced directional persistence on LM-332 compared to wild type cells (*P<0.01). (**C**) CD9/CD81si cells, but not CD151si cells, displayed reduced net distance traveled on LM-332 compared to wild type cells (*P<0.001). For A–C, values graphed are means ± s.e.m.; n = 12 trials (wild type), 9 trials (CD9/CD81si), and 3 trials (CD151si), with at least 25 cells of each type per trial. Data were analyzed by ANOVA with Dunnett's post test. (**D–F**) Motility of wild type, CD9/CD81si, and CD151si cells on collagen I. Migration velocity and net distance traveled of CD151si cells was significantly greater than wild type cells (*P<0.01, ANOVA with Dunnett post test). CD9/CD81si cell motility parameters were not different from wild type. Values graphed are mean ± s.e.m. for 25 cells of each type in one trial. (**G**) Wild type and CD151si cells displayed a similar number of rear protrusions on LM-332. Values graphed are mean ± s.e.m.; n = 3 trials with 25 cells of each type per trial.

The more severe spreading and adhesion defects in CD151si cells on LM-332 might have been anticipated, given that in CD151si cells, not only is CD151 virtually absent, but α3 association with the CD9/CD81 complex is also largely abrogated. However, analysis of post-spreading cell behaviors yielded unexpected results. In motility assays on LM-332 that were initiated after spreading was completed, the CD9/CD81si cells displayed reduced migration velocity, persistence, and net distance traveled. Surprisingly, the CD151si cells, once attached and spread, displayed wild type migration velocity, persistence, and net displacement (Fig. 4A–C). On collagen I, CD151si cells migrated significantly faster, and displayed significantly greater net distance traveled than either parental or CD9/CD81si cells (Fig. 4D, F). The directional persistence of CD9/81si cells appeared modestly reduced on collagen I, but the difference was not statistically significant (Fig. 4E). These results indicate that, although CD151 is critical for α3β1 integrin-dependent adhesion and spreading on LM-332, it appears less important for post-attachment migration in this system. Remarkably, the CD9/CD81 complex continues to be important for α3β1-dependent directed cell migration after cell spreading. This CD9/CD81 function might not involve a direct integrin association, since CD151si cells displayed wild type motility on LM-332, despite the fact that α3β1-CD9/CD81 association was disrupted in these cells (See Discussion). Consistent with the possible involvement of altered front-rear cell morphology in the CD9/CD81si cell migration phenotype, CD151si cells displayed a wild type number of tail retractions during migration on LM-332 (Fig. 4G).

**Figure 5:**
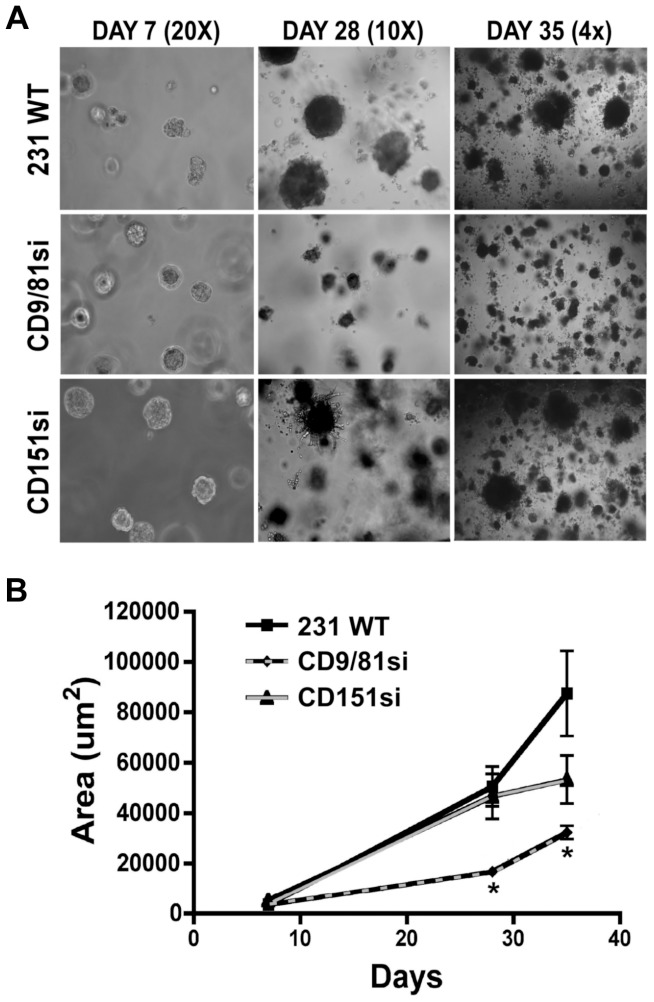
The CD9/CD81 complex promotes long-term growth of MDA-MB-231 cells in 3D Matrigel. (**A**) MDA-MB-231 wild type, CD9/CD81si, and CD151si cells were suspended in growth factor reduced Matrigel and imaged at 7, 28, and 35 d time points, using a 20×, 10×, and 4× objective respectively. (**B**) Average colony growth was calculated by measuring the total area for 20–34 colonies per time point using ImageJ software. Bars indicate mean ± s.e.m. CD9/CD81si colonies, but not CD151si colonies, were significantly smaller than wild type colonies at the 28 and 35 d time points (*P<0.01, ANOVA with Dunnett post test).

To further explore potential functional differences between CD151 and the CD9/CD81 complex, we next examined cell growth in 3D Matrigel, a behavior to which both α3 and α6 integrins are expected to contribute [28]. Over a 35 d assay, there was little apparent difference in colony size between parental and CD151si MDA-MB-231 cells, consistent with a previous report [28](Fig. 5A). In contrast, CD9/CD81si colony size was significantly reduced (Fig. 5A). Quantification revealed that CD151si and parental cell colonies were virtually identical through 28 d of growth, with CD151si colony size perhaps leveling off somewhat more by day 35 (Fig. 5B). In contrast, CD9/CD81si colonies were significantly smaller than parental colonies at all time points examined (Fig. 5B). These data provide another example of a cell behavior that is regulated differently by the CD9/CD81 complex than by CD151.

### α3β1 integrin-PKCα association is disrupted by depletion of CD9/CD81, but not by depletion of CD151 in MDA-MB-231 cells

**Figure 6:**
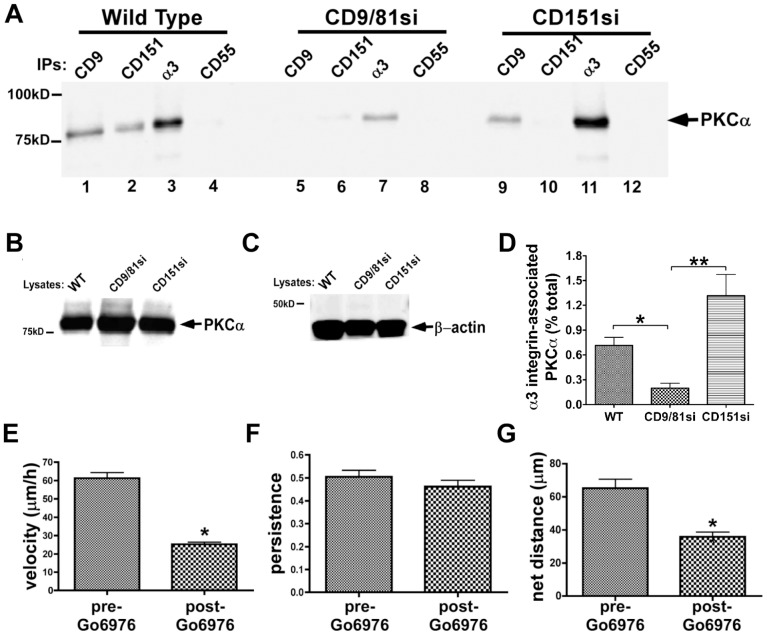
CD9/CD81 promotes PKCα-α3β1 integrin association, and PKCα promotes α3β1-dependent MDA-MB-231 cell motility. (**A**) MDA-MB-231 wild type, CD9/CD81si, and CD151si cells were treated with 100 nM PMA for 30 min, then lysed in 1% Brij 99 detergent followed by immunoprecipitation of CD9, CD151, α3 integrin, or CD55 and immunoblotting to detect PKCα. (**B,C**) Lysates of each cell type were immunoblotted for PKCα and β-actin. (**D**) The fraction of total cellular α3 integrin associated with PKCα in each cell type was estimated by LiCOR infrared fluorescence blot imaging, as described in Materials and Methods. Bars indicate mean ± S.E.M. from at least 3 different blots/cell type. CD9/CD81si cells showed a significant reduction in α3 integrin-associated PKCα compared to the other two cell types (*P<0.05, **P<0.01, ANOVA with Tukey post-test). (**E–G**) Wild type MDA-MB-231 cells were plated on LM-332-coated glass bottom dishes, and motility was monitored by time-lapse microscopy for 2 h before and 3 h after addition of PKC inhibitor Gö6976 (1 µM). Migration velocity and net distance traveled were both significantly reduced in the presence of Gö6976 (*P<0.0001, unpaired t test). Values graphed are means ± s.e.m. for 75 cells pre-Gö6976 and 70 cells post-Gö6976.

Tetraspanin proteins may regulate laminin-binding integrin function in part by promoting integrin association with activated classical protein kinase C (PKC) isoforms [37], [50], [56]–[58]. To explore potential PKC contributions to the phenotypes of our CD9/CD81si and CD151si cells, we first examined α3β1 integrin-PKCα association in these cells upon PMA stimulation. Preliminary experiments revealed that PMA-stimulation significantly enhances α3β1-PKCα association above basal levels (Fig. S4), as previously reported [50]. In Brij 99 detergent lysates, in which PKCα-α3β1 integrin association is preserved [50], PKCα co-precipitated with CD9, CD151, and α3 integrin, but not with the CD55 negative control in parental MDA-MB-231 cells (Fig. 6A, lanes 1–4). Co-precipitation of PKCα with CD9, CD151, and α3 integrin was dramatically reduced in the CD9/CD81si cell lysate (Fig. 6A, lanes 5–7). Surprisingly, in a CD151si cell lysate, co-precipitation of PKCα with both CD9 and α3 integrin was maintained at wild type levels (Fig. 6A, lanes 9 & 11), although it was lost from the CD151 immunoprecipitate, as expected (lane 10). Immunoblotting PKCα and β-actin in cell lysates confirmed that PKCα expression is unchanged in tetraspanin-silenced cells, and that similar amounts of total protein were input into each set of immunoprecipitations (Fig. 6B,C). Estimation of the fraction of total cellular PKCα that associates with α3β1 integrin in each cell type revealed an ∼75–85% reduction in α3β1-associated PKCα in the CD9/CD81si cells compared to parental or CD151si cells (Fig. 6D).

In a recent study, the contribution of CD151 to a PKCα-α6β4 integrin association was assayed in Brij 58, a milder detergent than Brij 99 [58]. To determine whether there might be a larger pool of PKCα that could associate with α3β1 integrin in a CD151-dependent manner in a milder detergent, we repeated our analysis using Brij 58 lysates. However, we obtained identical results in Brij 58 as in Brij 99 (Fig. S5).

To further confirm that CD151 expression is not essential for PKCα-α3β1 integrin association in MDA-MB-231 cells, we restored CD151 expression to the CD151si cells using an RNAi-resistant CD151 cDNA. Flow cytometry confirmed restoration of CD151 expression in these cells (CD151RX cells; Table I). Restoration of CD151 re-established α3β1 association with tetraspanin CD9 (Fig. S6A), and restored adhesion on LM-332 (Fig. S6B). Despite modulating association with CD9 down and then back up, neither silencing nor restoring CD151 expression reduced the amount of PKCα that co-precipitated with α3β1 integrin compared to that seen in wild type parental cells (Fig. S6C).

Collectively, these data indicate that CD151 is not required to promote PKCα-α3 integrin in MDA-MB-231 cells, but that the CD9/CD81 complex indeed plays an important role. The loss of PKCα-α3β1 association in the CD9/CD81si cells is not due to a gross alteration in the ability of PKCα to translocate to the plasma membrane, since we observed similar membrane localization of PKCα upon immunostaining either parental or CD9/CD81si cells (Fig. S7). Thus, while the mechanism by which the CD9/CD81 complex regulates PKCα-α3β1 association remains to be determined, our data indicate that it is likely to be more specific than a global disruption of PKCα's ability to associate with the plasma membrane.

Since both α3β1-dependent directed migration and α3β1-PKCα association were impaired in the CD9/CD81si cells, but not the CD151si cells, we next tested for a possible functional role of PKCα in α3β1-driven cell motility. Time-lapse video-microscopy revealed that upon addition of the classical PKC isoform inhibitor, Gö6976, parental MDA-MB-231 cells migrating on LM-332 slowed significantly and displayed a modest reduction in persistence, resulting in a substantial reduction in net distance traveled (Fig. 6D–F). Thus, an inhibitor of PKCα recapitulated key motility phenotypes observed in CD9/CD81si cells.

### The CD9/CD81 complex regulates α3β1 integrin-dependent motility in an alternate tumor cell system

To begin to investigate the generality of the role of the CD9/CD81 complex in regulating α3β1 function, we also created CD9/CD81-silenced A431 carcinoma cells. Flow cytometry confirmed that CD9 was 95% silenced and CD81 was 89% silenced in these cells (Table 1). Compared to wild type parental cells, the CD9/CD81si A431 cells displayed normal adhesion on LM-332 (data not shown), as we had observed in our MDA-MB-231 cells. However, in two separate trials, the CD9/CD81si A431 cells displayed significantly reduced migration velocity and net distance traveled (Fig. S8). There was also a trend towards reduced directional persistence that reached statistical significance in one trial, but not the other. Overall, these data indicate that the ability of the CD9/CD81 complex to promote α3β1 integrin-dependent motility is not restricted to the MDA-MB-231 breast cancer model, although, unlike MDA-MB-231 cells, A431 cells also exhibit an ongoing requirement for CD151 for rapid migration on LM-332 [30].

## Discussion

### Impact of CD9/CD81 depletion on α3β1 integrin function in tumor cells

The first major finding of our study is that the CD9/CD81 complex is in fact required for certain aspects of α3β1 integrin function in tumor cells. Numerous studies have documented that depleting or genetically ablating tetraspanin CD151 significantly impairs the functions of the laminin-binding α3 and α6 integrins [28], [30], [34]–[37], [44], [57]–[61]. However, far less had been known about the extent to which other tetraspanins, such as CD9 and CD81, are required for α3 or α6 integrin function.

MDA-MB-231 breast carcinoma cells depleted of CD9 were reported to display enhanced proliferation in 3D Matrigel (over a 5 day period), and reduced spreading (but enhanced chemotactic migration) on fibronectin [62]. CD9-silenced MDA-MB-231 cells also displayed reduced migration towards soluble, native collagen IV, a behavior that depended on discoidin domain receptor 1 [63]. In a recent study, an siRNA screen identified CD9 as a potent modulator of integrin function in transformed cells [64]. RNAi silencing of CD9 reduced Matrigel invasion of multiple tumor cell types, including PC-3 and 22Rv1 prostate carcinoma, and MDA-MB-231 breast carcinoma cells. Lung invasion by CD9-silenced MDA-MB-231 cells was also curtailed compared to control cells at 48 hours after tail vein injection [64]. While all of these studies pointed towards possible integrin-dependent functions for the CD9/CD81 complex in breast carcinoma cells, the specific integrins involved in each study were not defined, and, in particular, the specific contribution of α3β1 integrin was not assessed.

In addition to the MDA-MB-231 model, reported effects of silencing or genetically ablating CD9 or CD81 in other cell types include (i) reduced dendritic cell chemotactic migration on fibronectin [65], (ii) reduced small cell lung carcinoma cell adhesion on fibronectin, with increased apoptosis [66], (iii) reduced α2β1 integrin-dependent suppression of focal adhesion formation and cell proliferation for endothelial cells on LM-111 [67], (iv) reduced adhesion and migration of bone marrow-derived macrophage on Matrigel or fibronectin [68], (v) impaired αvβ5 integrin-dependent photoreceptor outer segment binding and engulfment by retinal pigmented epithelial cells [69], (vi) reduced expression of multiple β1 integrins and reduced cell spreading on Matrigel, LM-111, fibronectin, and collagen I in ovarian carcinoma cells [70], (vii) enhanced migration of bladder carcinoma cells on an unspecified substrate [71], and (viii) enhanced migration of primary melanocytes towards soluble fibronectin [72]. Again, all of these studies pointed towards the CD9/CD81 complex as a regulator of integrin-dependent cell behaviors, but in many of them, the specific integrins involved were not well-defined, and none of the studies focused on α3β1 integrin, a major tetraspanin partner.

Our new data now establish that depletion of the CD9/CD81 complex can have a significant impact on α3β1 integrin function in tumor cells. The loss of CD9 and CD81 delayed initial cell spreading and impaired directed cell motility on LM-332, two cell behaviors that we show here to strongly depend on α3β1 integrin but not on α6 integrins in MDA-MB-231 cells. Depletion of CD9 and CD81 had minimal impact on α2β1 integrin-dependent cell behaviors on collagen I, and re-expression of CD9 or CD81 reversed the phenotypes of CD9/CD81-silenced cells on LM-332. Thus, the impaired α3β1 functions in CD9/CD81si cells are due specifically to the loss of CD9 and CD81, and are unlikely due to off-target effects of RNAi or to a general reduction in cell motility. Similar results were obtained in A431 carcinoma cells, indicating that the ability of CD9/CD81 to regulate α3β1 integrin is not restricted to the MDA-MB-231 model.

### Overlapping but distinct effects of CD9/CD81 depletion versus CD151 depletion on α3β1 function in tumor cells

A surprising finding in our study was the degree of divergence between the phenotypes of the CD9/CD81-silenced MDA-MB-231 cells and the CD151-silenced cells. CD151 function has often been rationalized in terms of a model in which CD151 physically links laminin-binding integrins to other tetraspanins and TEM proteins. A simple prediction of this model might be that the impact on α3β1 function of depleting CD151 is likely to be more severe than the impact of depleting other subsets of TEM proteins, since the loss of CD151 should largely sever α3β1's physical association with all other TEM constituents. Indeed, in CD151-silenced MDA-MB-231 cells, α3β1 association with the CD9/CD81 complex is severely reduced. Moreover, in short term adhesion and spreading assays on LM-332, CD151-silenced cells were severely impaired, while the CD9/CD81-silenced cells showed a more modest reduction in spreading that did not translate to a measurable deficit in a simple adhesion assay. However, once cells were fully spread, assays of cell motility on LM-332 revealed an ongoing role for CD9/CD81, whereas CD151 appeared dispensable.

One potential explanation for this unexpected result is that during attachment and spreading, a smaller number of α3β1 receptors are initially involved in binding to the laminin substrate than the number of α3β1 receptors that are involved once the cells are fully attached and spread. Perhaps in the sub-optimal conditions that initially exist as cells first contact the laminin substrate, a physical association of α3β1 with fully intact TEMs is required for maximum function. Subsequently, when cells are fully spread and many more α3β1 receptors are engaged, association with fully intact TEMs may become less critical for α3β1 function in the MDA-MB-231 cell model. (A similar argument regarding receptor density has been made to explain the nominal effect of silencing CD151 on MCF-10A cell adhesion to LM-332 [37]). Nevertheless, even in fully spread cells, CD9/CD81 could continue to exert functions – perhaps independent of direct association with α3β1 – that are required for optimal α3β1-dependent motility. Our data indicate that such functions could include (i) helping to establish and maintain front-rear cell morphology and (ii) indirectly promoting PKCα association with α3β1. These two functions might be related, since cells expressing an α3 integrin mutant that lacks a cytoplasmic, PKC-controlled phosphorylation site display a reduction in tail retractions, reminiscent of what we observed for our CD9/CD81-silenced cells [56].

**Figure 7:**
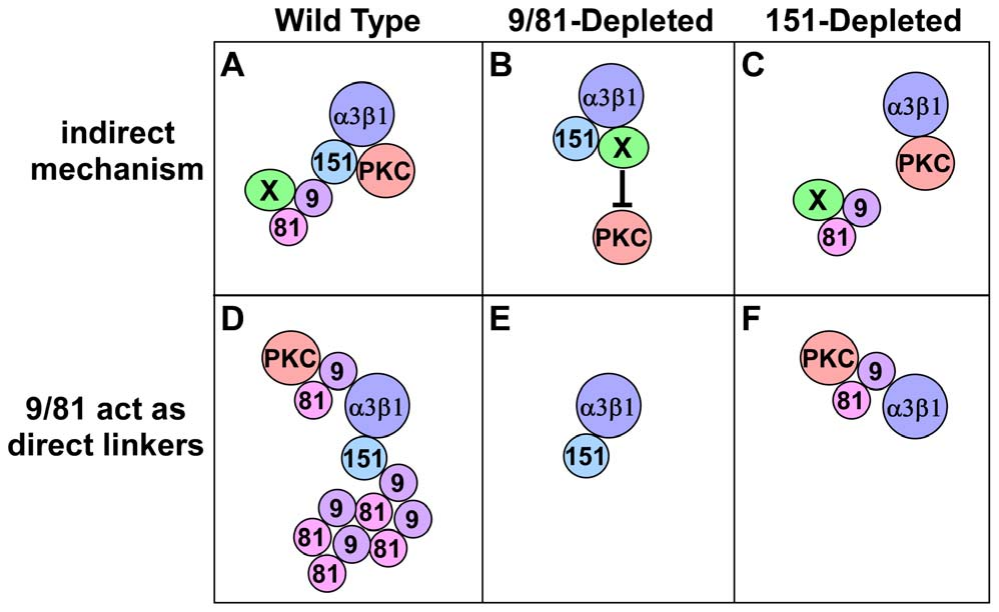
Hypothetical mechanisms of PKCα-α3β1 integrin association by the CD9/CD81 complex. (**A**) CD9/CD81 might regulate PKCα-α3β1 association indirectly, for example by sequestering a factor X that interferes with PKC's ability to interact with α3β1. (**B**) When CD9/CD81 are depleted, factor X might be released to block PKC-integrin association. (**C**) When CD151 is depleted, CD9/CD81 association with α3β1 is disrupted, but factor X remains sequestered, allowing PKC to continue to associate with α3β1. (**D**) A distinct pool of CD9/CD81 might act as a direct linker of PKC to α3β1 independently of CD151. (**E**) When CD9/CD81 are depleted, the linking function is disrupted, although CD151 remains associated with α3β1. (**F**) When CD151 is depleted, much of the CD9/CD81 is lost from the α3β1 complex, but that which remains is sufficient to link PKCα to α3β1.

In one hypothetical example of an indirect mechanism, CD9/CD81 could function by sequestering an unidentified factor X, which would otherwise inhibit the association of PKCα with α3β1 integrin. CD9/CD81 themselves can associate with α3β1 via CD151 in wild type cells (Fig. 7A), but CD9/CD81 continue to sequester the inhibitory factor X even when the CD9/CD81-α3β1 association is disrupted by the loss of CD151 (Fig. 7C). However, when CD9/CD81 are depleted, factor X is released to block PKCα-α3β1 association (Fig. 7B). Many other indirect mechanisms are possible, including completely indirect mechanisms where CD9/CD81 could influence signaling pathways that somehow regulate PKCα-α3β1 association independently of any CD9/CD81 association with other TEM proteins.

Another setting in which CD9/CD81 and CD151 functions diverged was in long term growth in 3D Matrigel. Compared to parental or CD151-silenced cells, CD9/CD81-silenced MDA-MB-231 cells displayed impaired growth over a 5 week assay. The minimal contribution of CD151 to growth in 3D Matrigel had been reported before for the MDA-MB-231 model [28]. Both α3 and α6 integrins can make contributions to MDA-MB-231 cell behavior in Matrigel [28], so whether CD9/CD81 contributions in this setting reflect regulation of α3 or α6 function (or both) remains to be determined.

### Potential involvement of PKCα in the pro-migratory functions of the CD9/CD81 complex

A third major finding uncovered in our study was the potential involvement of PKCα in CD9/CD81 pro-migratory functions. Congruent with the seemingly minimal contribution of CD151 to α3-dependent cell migration on LM-332, we also found that CD151 seems not to be required for α3β1 integrin-PKCα association in the MDA-MB-231 system. This result was also surprising, given that the ability of PKCβII to associate with α3β1 had previously been mapped to the α3 integrin *extracellular* domain [50]. This would seem to strongly implicate CD151 in linking α3β1 to classical PKC isoforms, since CD151 and α3 integrin engage in a direct, extracellular interaction. One potential explanation for the apparent discrepancy between our results and the previously published study of Zhang et al is that a residual CD9/CD81 association with α3β1 integrin in our CD151-silenced cells might be sufficient to mediate all of the α3β1-PKCα association. In this scenario, the bulk of CD9/CD81 would associate with α3β1 via linkage through CD151, but a much smaller pool would associate directly (Fig. 7D). Silencing CD151 abolishes most of the CD9/CD81 association with α3β1, but the small, directly interacting pool remains and is capable of mediating all the PKCα-α3β1 association (Fig. 7F). The hypothetical mechanism depicted in Fig. 7F may seem difficult to reconcile with our findings that CD9-α3β1 association appears virtually abolished in CD151-silenced cells. However, we and others have noted that α3β1 can retain low, residual association with other TEM-resident proteins upon near total loss of CD151 by RNAi, or even when CD151 is completely absent due to genetic deletion [35], [43], [44] (see also Fig. 3A, lane 9 and the middle lane of Fig. S6A in this study). Thus, although we currently favor an indirect mechanism, we cannot completely exclude the possibility that a residual CD9/CD81-α3β1 association in the CD151-silenced cells could directly mediate the PKCα-α3β1 association. We find it unlikely, however, that incomplete silencing of CD151 is responsible for preserving the wild type levels of PKCα-α3β1 association in our CD151-silenced cells. Flow cytometry revealed that CD151 expression was reduced by ∼99% in our CD151-silenced cells (Table I) with no impact on PKCα-α3β1 association. Yet silencing just 80–90% of CD9/CD81 was sufficient to reduce PKCα-α3β1 association by ∼75%. Thus, in the MDA-MB-231 system, the PKCα-α3β1 association appears to be much more sensitive to the total level of CD9/CD81 than to the amount of CD9/CD81 linked by CD151 to α3β1.

Another possibility is that the rules governing α3β1-PKCα association in adherent MDA-MB-231 cells differ from those that govern α3β1-PKCβII association in non-adherent K562 cells, which were used in the Zhang et al study [50]. For example, in the MDA-MB-231 system, the loss of CD151 may allow other as yet unidentified tetraspanins to associate directly with α3β1 integrin and recruit PKCα. Further experiments are required to clarify mechanism of α3β1-PKCα association in breast carcinoma cells.

The mechanism of α3β1-PKCα association may also differ from that of α6β4 integrin-PKCα association. In the MMTV-ErbB2 mouse breast cancer model, phosphorylation of the β4 integrin cytoplasmic tail at the PKC-dependent S1424 site was reduced in the primary mammary tumors from CD151 knockout mice versus control mice [57]. In addition, CD151-silenced MCF-10A breast cancer cells expressing ErbB2 also showed reduced β4 integrin phosphorylation at S1424 in response to EGF stimulation [57]. Similarly, phorbol ester or EGF-induced PKCα recruitment to α6β4, and PKC-dependent phosphorylation of the β4 cytoplasmic tail, were also significantly reduced in CD151-silenced A431 cells. However, PKCα-α6β4 association and β4 tail phosphorylation under basal conditions were unaffected by the loss of CD151, and CD151 was not absolutely required for integrin-PKCα association under any condition tested. Thus, while CD151 can clearly promote PKCα association with laminin-binding integrins in certain contexts, it appears not to be required as a critical linker in all cases. Consistent with CD9/CD81-dependent PKCα association playing an important role in α3β1 function, we showed that motility on LM-332 was significantly impaired upon treatment with the classical PKC isoform inhibitor Gö6976. Potential pro-migratory consequences of PKCα association with α3β1 include regulation of α3β1 cytoskeletal association via PKC-controlled phosphorylation of the α3 integrin cytoplasmic tail [56], and, by analogy to α6 integrin, regulation of α3β1 diffusion mode [37].

### Conclusion

PKCα is emerging as a potential promoter of tumor progression and metastasis. One recent study showed that a novel, PKCα inhibitory peptide can strongly inhibit spontaneous metastasis in the 4T1 murine mammary carcinoma cell model [73]. In addition, PKCα expression level correlates with estrogen receptor/progesterone receptor-negativity, higher tumor grade, increased Ki-67 positivity, and poor prognosis in human breast cancer [74], and the metastasis inhibitor KiSS1 may function in part by downregulating PKCα [75]. A recent study has also revealed a critical role for α3β1 in MDA-MB-231 breast carcinoma tumor formation in vivo [22]. Together with these studies, our new data raise the distinct possibility that the pro-metastatic, pro-tumor activity of PKCα in breast cancer may derive in part from PKCα's ability to promote α3β1 integrin-dependent tumor cell motility. If PKCα does promote breast cancer metastasis through α3β1 integrin, our new data strongly suggest that the CD9/CD81 complex plays a key role. Since CD151 also promotes metastatic colonization in the MDA-MB-231 model [36] (but seems not to be required for PKCα association with α3β1 in this model), simultaneously inhibiting both CD9/CD81 and CD151 functions might have an even more profound inhibitory effect on metastasis. Future studies should aim to elucidate the mechanism by which PKCα regulates α3β1 function in tumor cells, and the interplay between CD9/CD81, CD151, and other TEM-resident proteins such as the major CD9/CD81 partners, IgSF proteins EWI-2 and EWI-F/CD9P-1, in regulating metastatic cell behaviors.

## Supporting Information

Figure S1 **Normal Cell Spreading and Cell Migration on Laminin-332 in MDA-MB-231 CD9 or CD81 single mutants.** (**A**) MDA-MB-231 wild type, CD9si, and CD81si cells were plated on LM-332-coated glass coverslips, and cell spreading was imaged 30 min later by phase contrast microscopy. The area of cell spreading for each cell type was calculated by subtracting the mean area of cells imaged immediately after plating from the mean area of cells after 30 min of spreading. Values graphed are means ± s.e.m.; n = 3 trials with at least 25 cells of each type per trial. (**B**) MDA-MB-231 wild type, CD9si, and CD81si cells were plated on LM-332-coated glass bottom dishes, and cell motility was monitored for 3 h by time-lapse microscopy. Values graphed are means ± s.e.m; n = 2 trials with 64–84 cells of each type per trial.
(TIF)

Figure S2 **MDA-MB-231 cell responses to LM-332 are strongly α3β1 integrin-dependent.** (**A**) MDA-MB-231 cells were left untreated or were treated with 10 µg/ml of A3-IIF5 anti-α3 integrin antibody, 10 µg/ml GoH3 anti-α6 integrin antibody, or both antibodies for 10 min prior to plating on LM-332. After 30 min cells were fixed and imaged. (**B**) MDA-MB-231 cells were plated on LM-332, and motility was monitored by time-lapse video-microscopy for 2 h. Then A3-IIF5 anti-α3 integrin or GoH3 anti-α6 integrin function blocking antibody was added to the cells at 10 µg/ml, and migration was observed for an additional 2 hours. The graph shows the cell migration velocity for each treatment condition. The values are means ± s.e.m.; * p<0.01 compared with WT untreated cells (student's t-test). Each column represents the average from 30–50 individually migrating cells.
(TIF)

Figure S3 **Quantification of leading edge cortactin.** The number of wild type and CD9/CD81si cells with or without cortactin at the leading edge was quantified by scoring 105 wild type cells and 202 CD9/CD81si cells as positive or negative for leading edge cortactin. In wild type cells 88/105 cells (84%) had leading edge cortactin, while in CD9/CD81si cells, only 48/202 cells (24%) had leading edge cortactin. This difference is significant P<0.0001 by two sided Fisher's exact test.
(TIF)

Figure S4 **PMA stimulates PKCα association with α3β1 integrin and CD9.** MDA-MB-231 cells were left untreated (**A**) or treated with 100 nM PMA (**B**) for 30 min prior to lysis in 1% Brij 99. CD9, CD151, α3 integrin, or CD55 were immunoprecipitated followed by blotting for PKCα. (**C**) Blotting PKCα in lysates of untreated or PMA-treated cells revealed similar total levels of extractable PKCα under both conditions.
(TIF)

Figure S5 **Requirement for the CD9/CD81 complex, but not CD151, in mediating the PKCα-α3β1 integrin association in milder Brij 58 lysis conditions.** (**A**) MDA-MB-231 wild type, CD9/CD81si, and CD151si cells were lysed in 1% Brij 58 detergent followed by immunoprecipitation of CD9, CD151, α3 integrin, or CD55 and immunoblotting to detect PKCα. (**B,C**) Lysates of each cell type were also blotted for PKCα or β-actin.
(TIF)

Figure S6 **Re-expression of CD151 in the CD151si MDA-MB-231 cells.** An RNAi-resistant CD151 cDNA was introduced into CD151si cells to create CD151RX cells. (**A**) CD9 was immunoprecipitated from 1% Brij 96V/Brij99 lysates of wild type, CD151si, and CD151RX cells, followed by immunoblotting with A3-CYT anti-α3 integrin antibody. (**B**) Wild type, CD151si, and CD151Rx were used for an adhesion assay on laminin-332 as in Fig. 3B. Bars represent mean ± S.E.M. for 4 wells/cell type. CD151si cell adhesion was significantly lower than wild type or CD151RX cell adhesion (*P<0.001, ANOVA with Tukey post-test). (**C**) Integrin α3 was immunoprecipitated from lysates of PMA-stimulated cells, and the amount of PKCα co-precipitating with α3β1 integrin in wild type, CD151si, and CD151RX cells was quantified using LI-COR Studio Lite software.
(TIF)

Figure S7 **PKCα localization in MDA-MB-231 wild type and CD9/CD81si cells.** Cells plated on LM-332 were stimulated or not with PMA for 30 minutes, and then fixed, permeabilized, and stained with anti-PKCα antibody SC208 (Santa Cruz), followed by Alexa-488 goat-anti-rabbit secondary antibody. Loss of CD9/CD81 does not prevent PKCα from localizing to ruffling edges under either basal or PMA-stimulated conditions.
(TIF)

Figure S8 **The CD9/CD81 complex regulates α3β1 integrin-dependent motility in A431 epidermoid carcinoma cells.** A431 wild type and CD9/CD81si cells were plated on LM-332-coated glass bottom dishes, and cell motility was monitored for 3 h by time-lapse microscopy. Values graphed are means ± s.e.m.; n≥50 cells of each type per experiment. The CD9/CD81si cells showed impaired cell migration parameters (*P<0.001 in A,B,&C; P = 0.0084 in D; P = 0.02 in F, unpaired t test).
(TIF)
