# Supplementary figures and images for: The CD9/CD81 Tetraspanin Complex and Tetraspanin CD151 Regulate α3β1 Integrin-Dependent Tumor Cell Behaviors by Overlapping but Distinct Mechanisms
Source: PLoS One. 2013 Apr 17;8(4):e61834. doi: 10.1371/journal.pone.0061834 (PMC3629153; doi:10.1371/journal.pone.0061834)

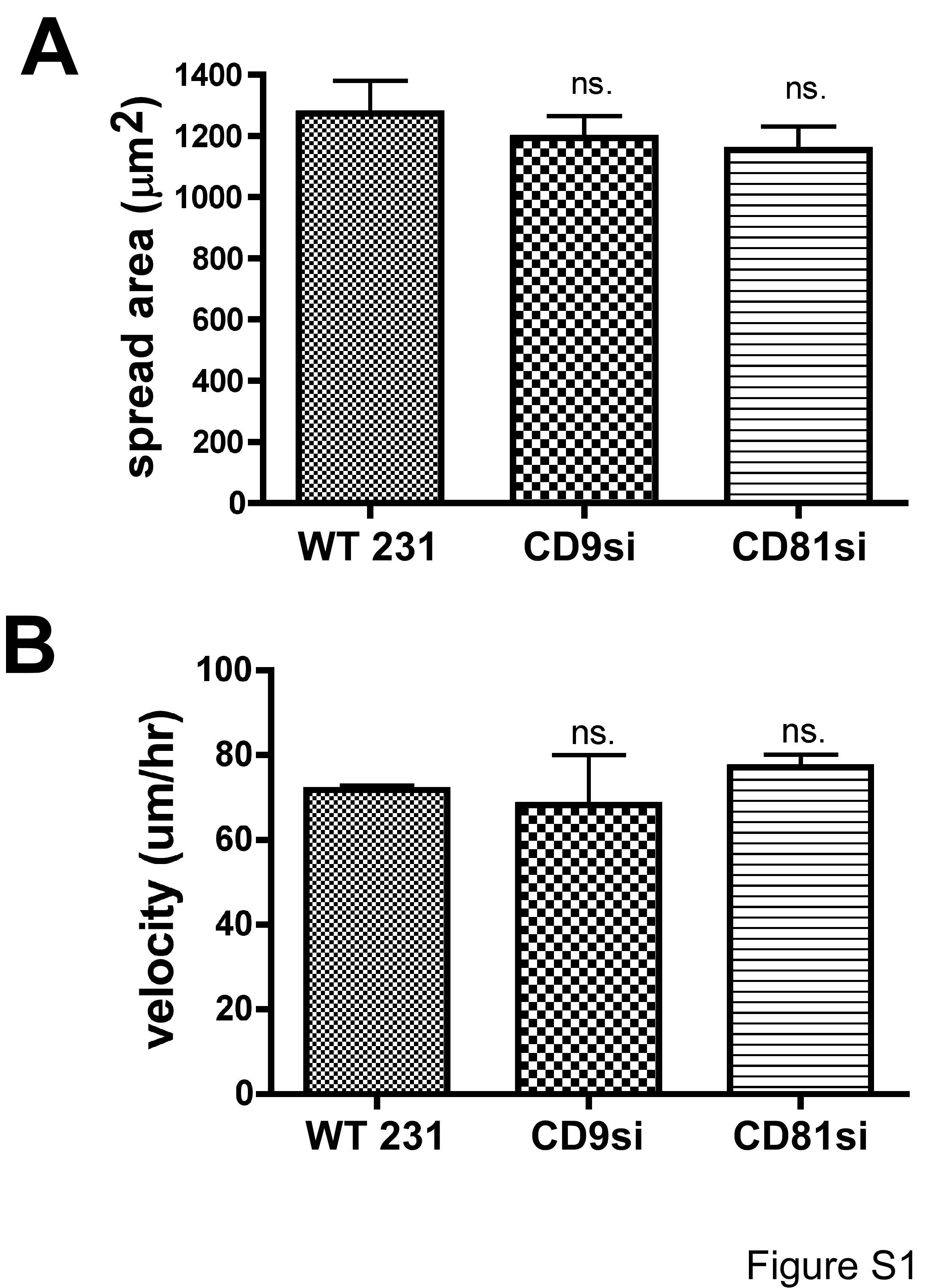

Supplement: Figure S1 — Normal Cell Spreading and Cell Migration on Laminin-332 in MDA-MB-231 CD9 or CD81 single mutants. (A) MDA-MB-231 wild type, CD9si, and CD81si cells were plated on LM-332-coated glass coverslips, and cell spreading was imaged 30 min later by phase contrast microscopy. The area of cell spreading for each cell type was calculated by subtracting the mean area of cells imaged immediately after plating from the mean area of cells after 30 min of spreading. Values graphed are means ± s.e.m.; n = 3 trials with at least 25 cells of each type per trial. (B) MDA-MB-231 wild type, CD9si, and CD81si cells were plated on LM-332-coated glass bottom dishes, and cell motility was monitored for 3 h by time-lapse microscopy. Values graphed are means ± s.e.m; n = 2 trials with 64–84 cells of each type per trial. (TIF) [file pone.0061834.s001.tif]

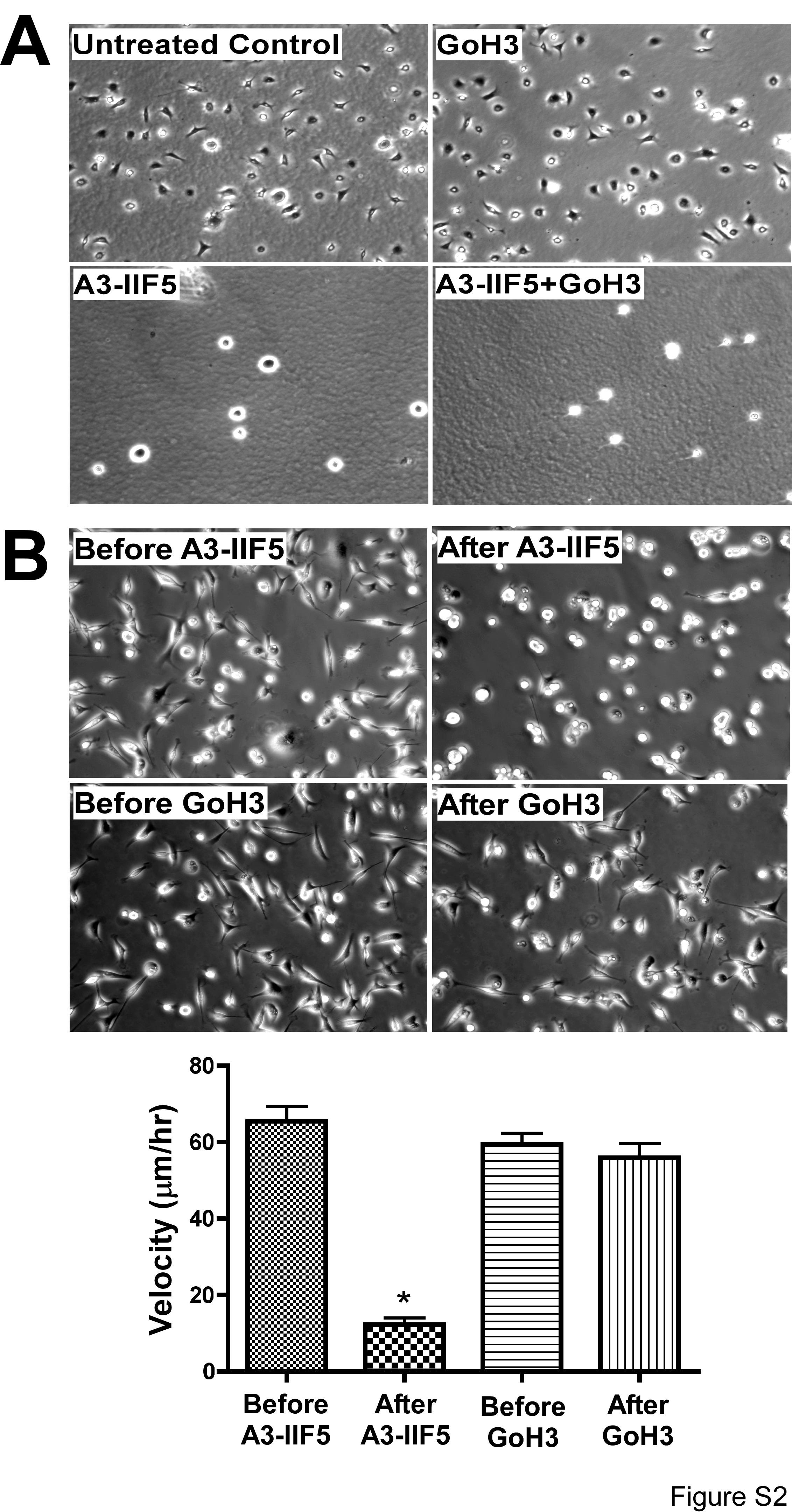

Supplement: Figure S2 — MDA-MB-231 cell responses to LM-332 are strongly α3β1 integrin-dependent. (A) MDA-MB-231 cells were left untreated or were treated with 10 µg/ml of A3-IIF5 anti-α3 integrin antibody, 10 µg/ml GoH3 anti-α6 integrin antibody, or both antibodies for 10 min prior to plating on LM-332. After 30 min cells were fixed and imaged. (B) MDA-MB-231 cells were plated on LM-332, and motility was monitored by time-lapse video-microscopy for 2 h. Then A3-IIF5 anti-α3 integrin or GoH3 anti-α6 integrin function blocking antibody was added to the cells at 10 µg/ml, and migration was observed for an additional 2 hours. The graph shows the cell migration velocity for each treatment condition. The values are means ± s.e.m.; * p<0.01 compared with WT untreated cells (student's t-test). Each column represents the average from 30–50 individually migrating cells. (TIF) [file pone.0061834.s002.tif]

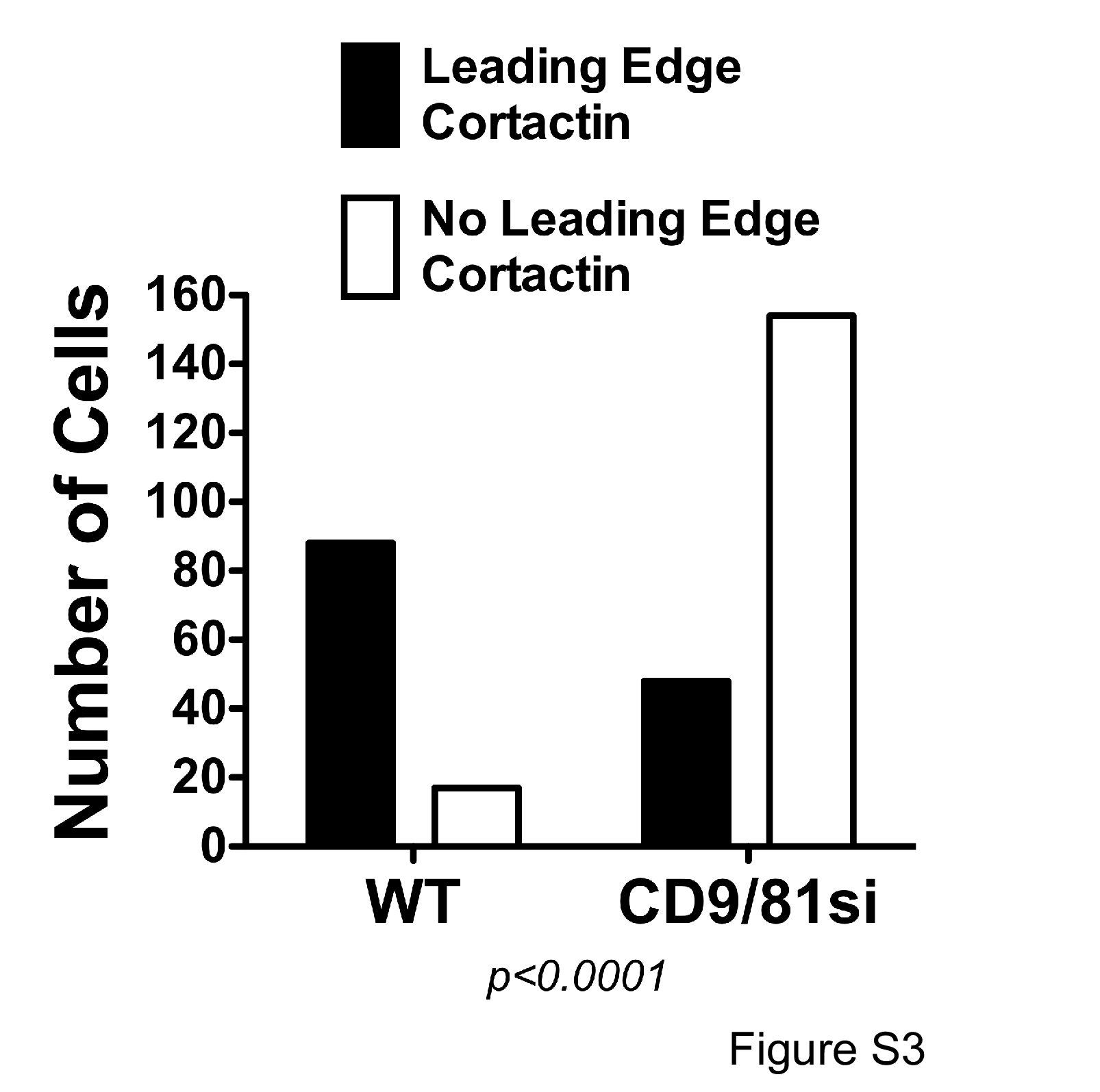

Supplement: Figure S3 — Quantification of leading edge cortactin. The number of wild type and CD9/CD81si cells with or without cortactin at the leading edge was quantified by scoring 105 wild type cells and 202 CD9/CD81si cells as positive or negative for leading edge cortactin. In wild type cells 88/105 cells (84%) had leading edge cortactin, while in CD9/CD81si cells, only 48/202 cells (24%) had leading edge cortactin. This difference is significant P<0.0001 by two sided Fisher's exact test. (TIF) [file pone.0061834.s003.tif]

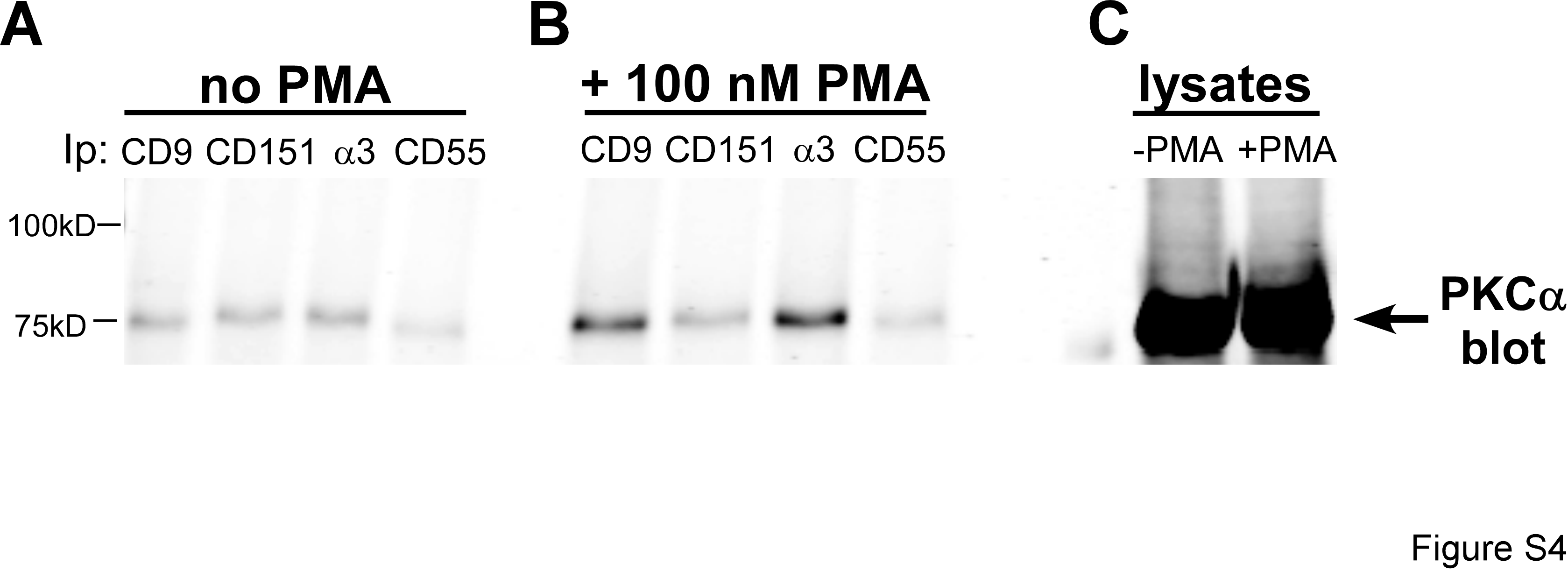

Supplement: Figure S4 — PMA stimulates PKCα association with α3β1 integrin and CD9. MDA-MB-231 cells were left untreated (A) or treated with 100 nM PMA (B) for 30 min prior to lysis in 1% Brij 99. CD9, CD151, α3 integrin, or CD55 were immunoprecipitated followed by blotting for PKCα. (C) Blotting PKCα in lysates of untreated or PMA-treated cells revealed similar total levels of extractable PKCα under both conditions. (TIF) [file pone.0061834.s004.tif]

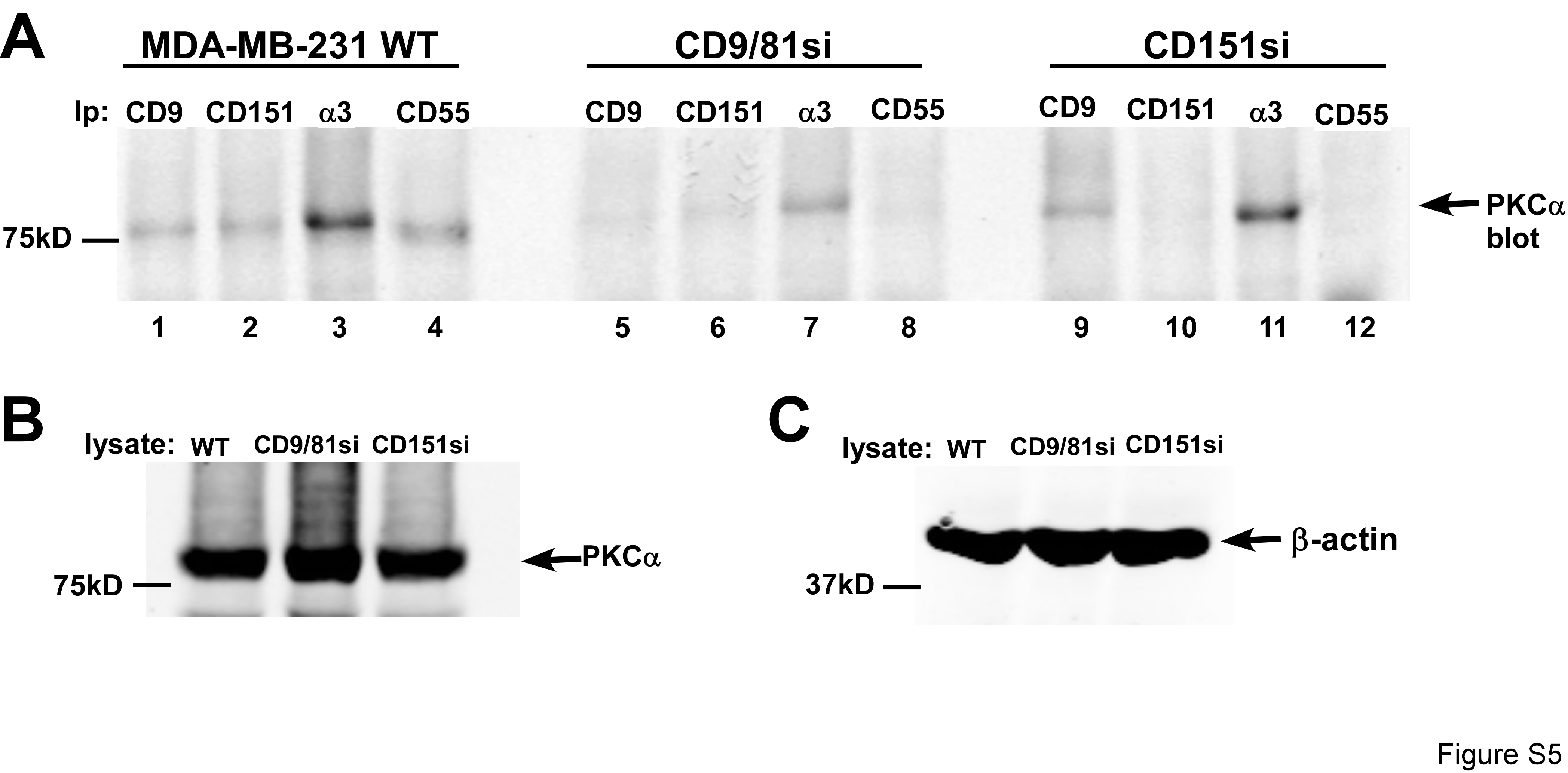

Supplement: Figure S5 — Requirement for the CD9/CD81 complex, but not CD151, in mediating the PKCα-α3β1 integrin association in milder Brij 58 lysis conditions. (A) MDA-MB-231 wild type, CD9/CD81si, and CD151si cells were lysed in 1% Brij 58 detergent followed by immunoprecipitation of CD9, CD151, α3 integrin, or CD55 and immunoblotting to detect PKCα. (B,C) Lysates of each cell type were also blotted for PKCα or β-actin. (TIF) [file pone.0061834.s005.tif]

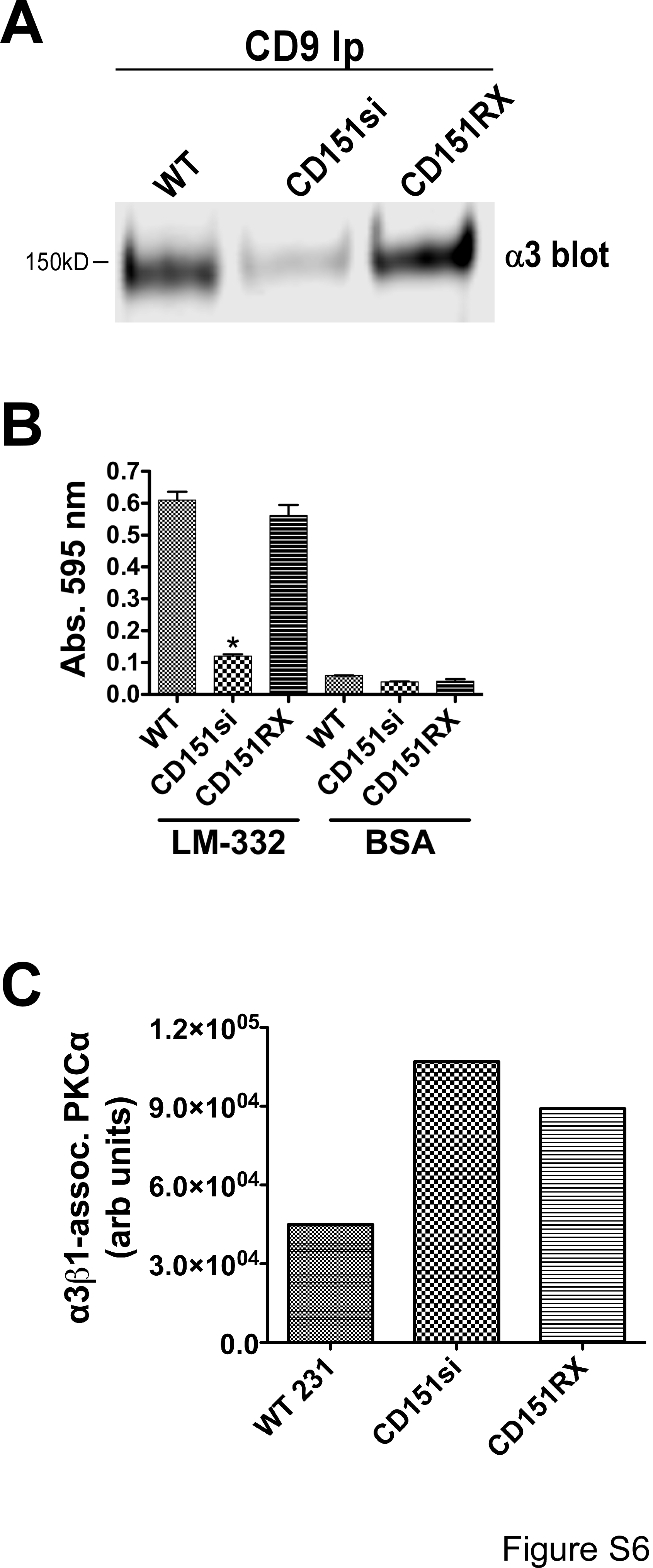

Supplement: Figure S6 — Re-expression of CD151 in the CD151si MDA-MB-231 cells. An RNAi-resistant CD151 cDNA was introduced into CD151si cells to create CD151RX cells. (A) CD9 was immunoprecipitated from 1% Brij 96V/Brij99 lysates of wild type, CD151si, and CD151RX cells, followed by immunoblotting with A3-CYT anti-α3 integrin antibody. (B) Wild type, CD151si, and CD151Rx were used for an adhesion assay on laminin-332 as in Fig. 3B. Bars represent mean ± S.E.M. for 4 wells/cell type. CD151si cell adhesion was significantly lower than wild type or CD151RX cell adhesion (*P<0.001, ANOVA with Tukey post-test). (C) Integrin α3 was immunoprecipitated from lysates of PMA-stimulated cells, and the amount of PKCα co-precipitating with α3β1 integrin in wild type, CD151si, and CD151RX cells was quantified using LI-COR Studio Lite software. (TIF) [file pone.0061834.s006.tif]

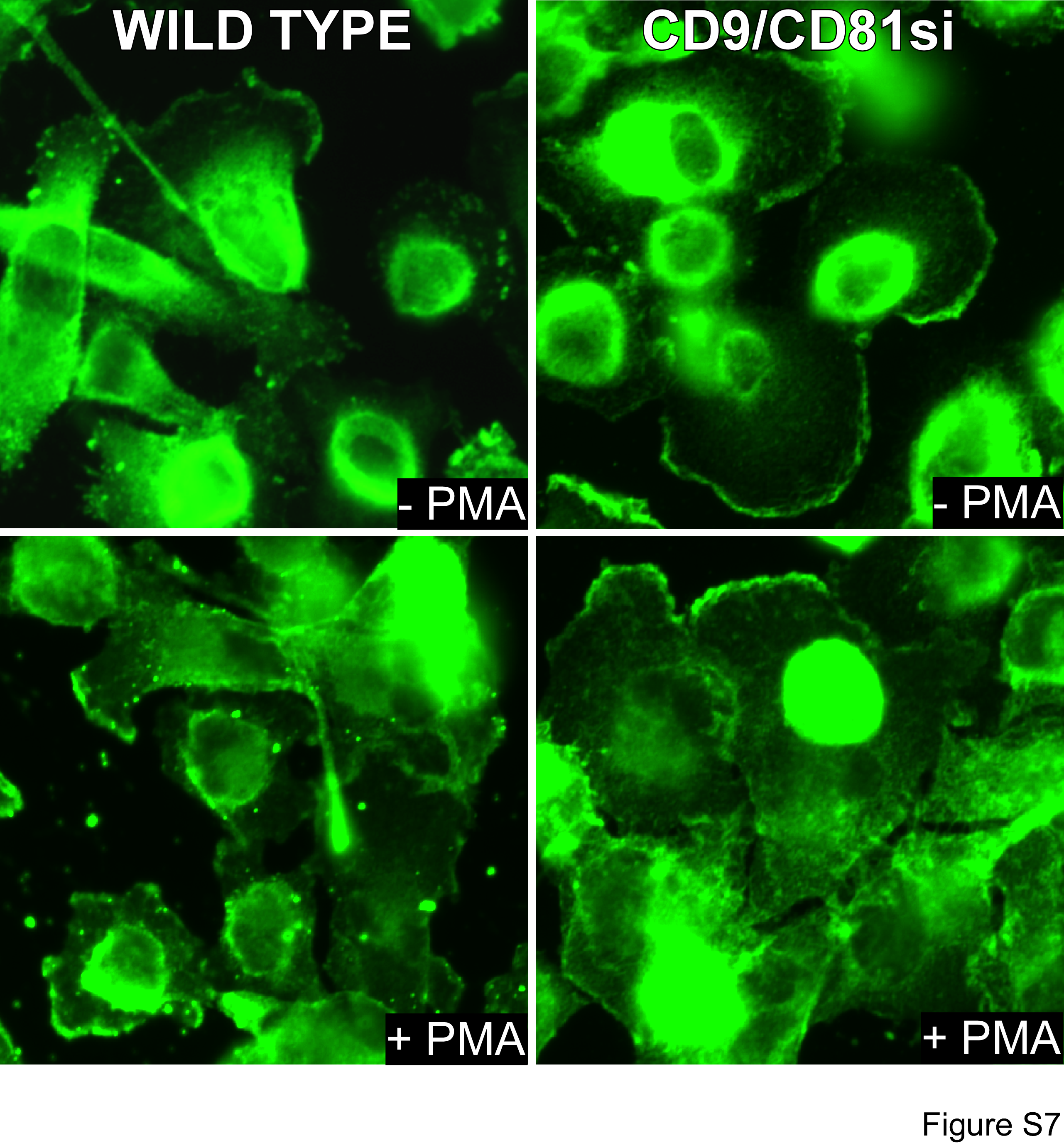

Supplement: Figure S7 — PKCα localization in MDA-MB-231 wild type and CD9/CD81si cells. Cells plated on LM-332 were stimulated or not with PMA for 30 minutes, and then fixed, permeabilized, and stained with anti-PKCα antibody SC208 (Santa Cruz), followed by Alexa-488 goat-anti-rabbit secondary antibody. Loss of CD9/CD81 does not prevent PKCα from localizing to ruffling edges under either basal or PMA-stimulated conditions. (TIF) [file pone.0061834.s007.tif]

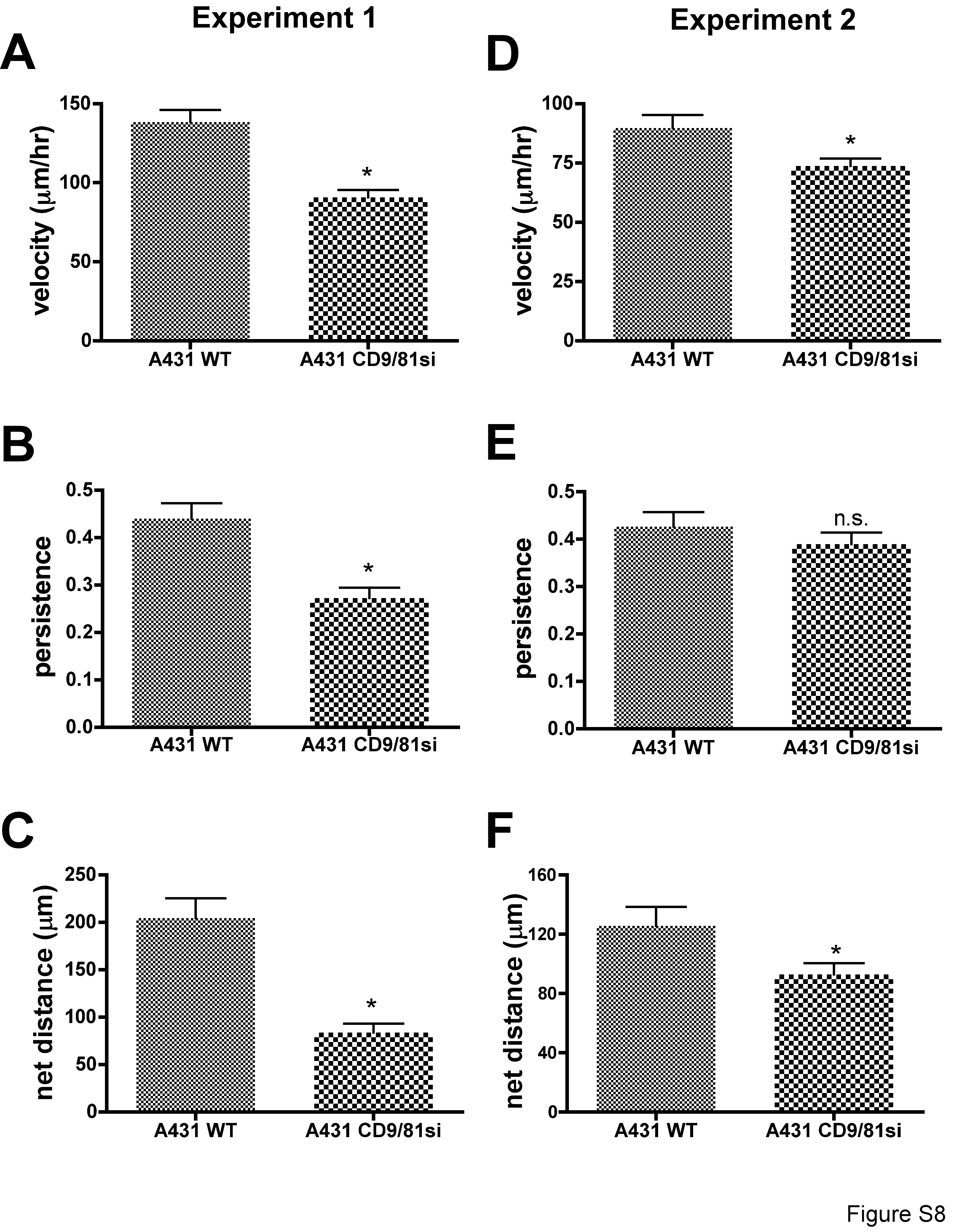

Supplement: Figure S8 — The CD9/CD81 complex regulates α3β1 integrin-dependent motility in A431 epidermoid carcinoma cells. A431 wild type and CD9/CD81si cells were plated on LM-332-coated glass bottom dishes, and cell motility was monitored for 3 h by time-lapse microscopy. Values graphed are means ± s.e.m.; n≥50 cells of each type per experiment. The CD9/CD81si cells showed impaired cell migration parameters (*P<0.001 in A,B,&C; P = 0.0084 in D; P = 0.02 in F, unpaired t test). (TIF) [file pone.0061834.s008.tif]
